# RNA 3D structure prediction guided by independent folding of homologous sequences

**DOI:** 10.1186/s12859-019-3120-y

**Published:** 2019-10-22

**Authors:** Marcin Magnus, Kalli Kappel, Rhiju Das, Janusz M. Bujnicki

**Affiliations:** 1 Laboratory of Bioinformatics and Protein Engineering, International Institute of Molecular and Cell Biology, Warsaw, Poland; 2 Biophysics Program, Stanford University, Stanford, CA USA; 3 Department of Biochemistry, Stanford University, Stanford, CA USA; 4 Department of Physics, Stanford University, Stanford, CA USA; 5 Laboratory of Bioinformatics, Institute of Molecular Biology and Biotechnology, Adam Mickiewicz University, Poznan, Poland

**Keywords:** RNA, RNA 3D structure prediction, RNA folding, RNA evolution, Rosetta, SimRNA

## Abstract

**Background:**

The understanding of the importance of RNA has dramatically changed over recent years. As in the case of proteins, the function of an RNA molecule is encoded in its tertiary structure, which in turn is determined by the molecule’s sequence. The prediction of tertiary structures of complex RNAs is still a challenging task.

**Results:**

Using the observation that RNA sequences from the same RNA family fold into conserved structure, we test herein whether parallel modeling of RNA homologs can improve ab initio RNA structure prediction. EvoClustRNA is a multi-step modeling process, in which homologous sequences for the target sequence are selected using the Rfam database. Subsequently, independent folding simulations using Rosetta FARFAR and SimRNA are carried out. The model of the target sequence is selected based on the most common structural arrangement of the common helical fragments. As a test, on two blind RNA-Puzzles challenges, EvoClustRNA predictions ranked as the first of all submissions for the L-glutamine riboswitch and as the second for the ZMP riboswitch. Moreover, through a benchmark of known structures, we discovered several cases in which particular homologs were unusually amenable to structure recovery in folding simulations compared to the single original target sequence.

**Conclusion:**

This work, for the first time to our knowledge, demonstrates the importance of the selection of the target sequence from an alignment of an RNA family for the success of RNA 3D structure prediction. These observations prompt investigations into a new direction of research for checking 3D structure “foldability” or “predictability” of related RNA sequences to obtain accurate predictions. To support new research in this area, we provide all relevant scripts in a documented and ready-to-use form. By exploring new ideas and identifying limitations of the current RNA 3D structure prediction methods, this work is bringing us closer to the near-native computational RNA 3D models.

## Background

Ribonucleic acid (RNA) is one of the key types of molecules found in living cells. It is involved in a number of highly important biological processes, not only as the carrier of the genetic information but also serving catalytic, scaffolding and structural functions, and more [1]. The interest in the field of non-coding RNA such as circular RNAs [2], long non-coding RNAs [3] has been increasing for the past few decades with new types of non-coding RNAs discovered every year. Similarly to proteins, a 3D structure of an RNA molecule determines its function. In order to build a 3D model of an RNA particle, one can take advantage of high-resolution experimental techniques, such as biocrystallography [4, 5], cryo-EM [6], and nuclear magnetic resonance spectroscopy [7]. However, experimental techniques are tedious, time-consuming, expensive, require specialized equipment, and not always can be applied. An alternative and complement to experimental techniques are methods for computational modeling. However, the results of the RNA-Puzzles [8, 9], a collective experiment for RNA structure prediction, show that while accurate modeling of RNA is achievable, there is still room for improvement. In particular, recent tests [10] have demonstrated significant progress. Although encouraging, this progress still leaves the field without methods that can reliably predict RNA tertiary structure in a consistent way.

Just like proteins, RNAs can be grouped into families [11] that have evolved from a common ancestor. Sequences of RNAs from the same family can be aligned to each and equivalency at the level of individual residues can be represented by a multiple sequence alignment (MSA). The analysis of patterns of sequence conservation or the lack thereof can be used to detect important conserved regions, e.g., regions that bind ligands, active sites, or are involved in other important functions. An accurate RNA sequence alignment can be used to predict secondary structure, the Watson-Crick base pairing pattern for the RNA, a key precedent for subsequently modeling RNA tertiary structure. According to the CompaRNA [12] continuous benchmarking platform, methods that exploit RNA alignments, such as PETfold [13] outperform single sequence predictive methods for RNA secondary structure.

RNA alignments can be used to improve tertiary structure prediction. Weinreb and coworkers [14] adapted the maximum entropy model to RNA sequence alignments to predict long-range contacts between residues for 180 RNA gene families. They applied the information about predicted contacts to guide in silico simulations and observed significant improvement in predictions of five cases they researched. Another method was proposed by Martin Weigt’s group [15]. These methods are reviewed elsewhere [16].

**Fig. 1:**
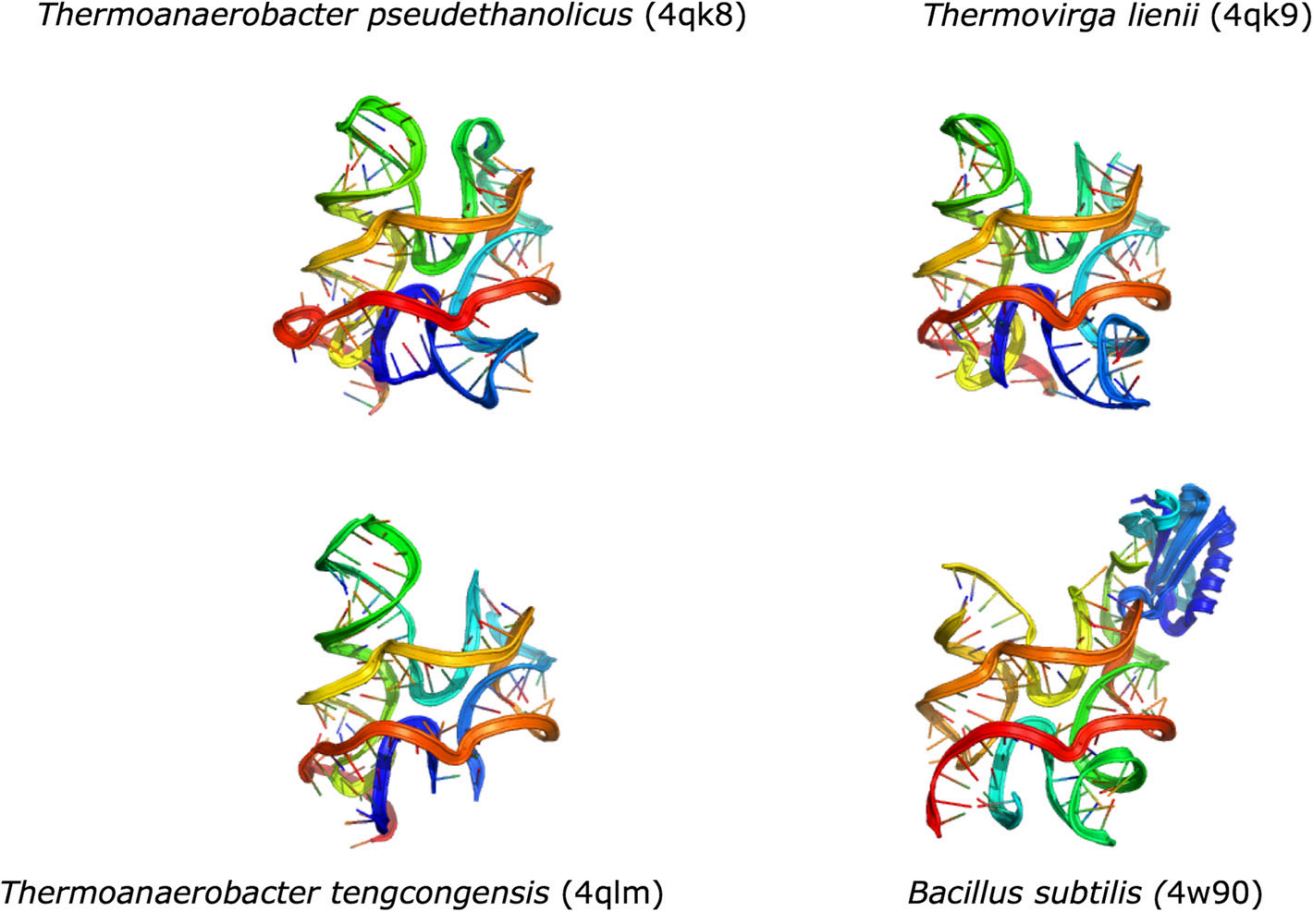
RNA families tend to fold into the same 3D shape. Structures of the riboswitch c-di-AMP solved independently by three groups: for two different sequences obtained from *Thermoanaerobacter pseudethanolicus* (PDB ID: 4QK8) and *Thermovirga lienii* (PDB ID: 4QK9) [18] for a sequence from *Thermoanaerobacter tengcongensis (PDB ID: 4QLM)* [19] and for a sequence from *Bacillus subtilis* (PDB ID: 4 W90) (the molecule in blue is a protein used to facilitate crystallization) [20]. There is some variation between structures in the peripheral parts, but the overall structure of the core is conserved

In this work, a distinct way to use RNA alignment for tertiary structure prediction is investigated. The proposed approach explores the use of multiple sequence alignment information and parallel modeling of RNA homologs to improve ab initio RNA structure prediction method. A new approach, named EvoClustRNA, takes advantage of incorporation of evolutionary information from distant sequence homologs and is based on a classic strategy of protein structure prediction [17]. By building on the empirical observation that RNA sequences from the same RNA family typically fold into similar 3D structures (Fig. 1), we tested whether it is possible to guide in silico modeling by seeking a global helical arrangement, for the target sequence, that is shared across de novo models of numerous sequence homologs. To the best of our knowledge, EvoClustRNA is the first attempt to use this approach for RNA 3D structure prediction.

We tested the EvoClustRNA coupled with two RNA 3D structure prediction methods, SimRNA [21] and Rosetta FARFAR (fragment assembly of RNA with full-atom refinement) [22]. SimRNA uses a coarse-grained representation, relies on the Monte Carlo method for sampling the conformational space, and employs a statistical potential to approximate the energy and identify conformations that correspond to biologically relevant structures. Similarly, Rosetta FARFAR uses coarse-grained representation and the Monte Carlo sampling. The main difference between the methods is how the simulation is performed. SimRNA starts from an unfolded conformation of an RNA molecule and runs a replica-exchange Monte Carlo simulation to fold it. By contrast, Rosetta builds initial conformations using a library of fragments and performs the Monte Carlo sampling to generate a low-resolution model. This procedure is repeated to obtain 10,000–20,000 models. The models can then be further refined in an all-atom potential to yield more realistic structures.

We also describe the usage of a tool that we have developed for clustering visualization named Clanstix. The tool allowed to understand the relationship between models for various homologs and reference structures.

Moreover, we report tests in the RNA-Puzzles 13 and 14 blind modeling trials, systematic benchmarking of the approach, and a description of the automated workflow that is now made available for the research community.

## Results

### EvoClustRNA workflow

In this work, we propose a new methodology together with ready-to-use implementation (EvoClustRNA), that can contribute to the improvement of RNA 3D structure prediction. The EvoClustRNA method takes as input (i) an alignment file, (ii) a folder with models generated for homologous sequence, and (iii) a file that maps sequence names from the alignment with filenames of models.

**Fig. 2:**
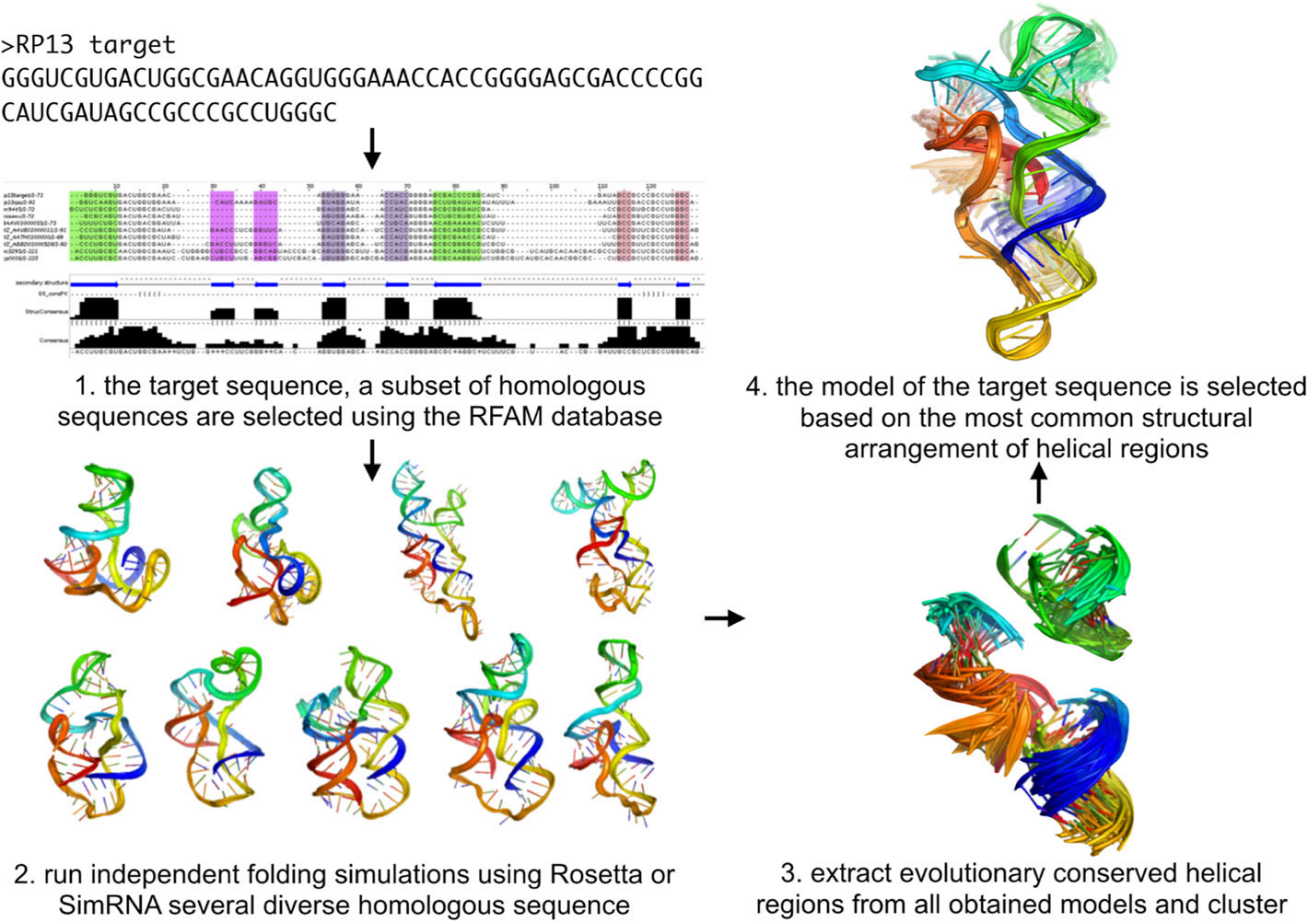
The workflow implemented as EvoClustRNA - as an example of a structure prediction of the ZMP Riboswitch (RNA-Puzzle 13). (1) Sequences of homologs are found for the target sequence, and an RNA alignment is prepared. (2) Using Rosetta and/or SimRNA structural models for all sequences are generated. (3) The conserved regions are extracted and clustered. (4) The final prediction of the method is the model containing the most commonly preserved structural arrangements in the set of homologs

The input preparation for the workflow has to be performed manually by the user (Fig. 2. 1–2). An input alignment can be obtained from the Rfam database or generated by the user. Sequences in the alignment should be sorted by length, and the redundancy removal procedure should be applied to remove similar sequences. In the proposed protocol, the shortest homologs are modeled using the SimRNAweb server or/and Rosetta. At the final stage of the input preparation, the top 100 models from a simulation should be moved to the input folder for the EvoClustRNA workflow.

We recommend to fold the shortest homologs because the average accuracy of de novo prediction of RNA 3D structure deteriorates with the increased length of RNA (e.g., [10, 23]). The volume of the conformational space that needs to be sampled grows exponentially with the chain length [24, 25]. Furthermore, de novo structure prediction methods rely on multiple approximations (e.g., coarse-grained representations, crude statistical potentials) thus with the increased size of the system under study small errors accumulate. Moreover, the computational cost increases with the molecule size for the calculation of energy for each conformation, which also increases the computational cost for a fixed simulation length.

When the input files are ready, the next step of the process (Fig. 2. 3–4) can be executed. The EvoClustRNA package contains tools to make the process as easy as possible, starting from processing input models to obtain all-vs-all core RMSD matrix (evoClustRNA.py), automated clustering procedure (evoClust_autoclustix.py), ending with a script to calculate the accuracy of prediction (evoClust_calc_rmsd.py). The model of the target sequence with the highest number of neighbors is selected as the final prediction.

The full workflow can be accessed at GitHub https://github.com/mmagnus/EvoClustRNA with the use cases, e.g., for the RNA-Puzzle 13 (https://github.com/mmagnus/EvoClustRNA/tree/master/test_data/rp13).

### Blind predictions with EvoClustRNA in the RNA-Puzzles

**Fig. 3:**
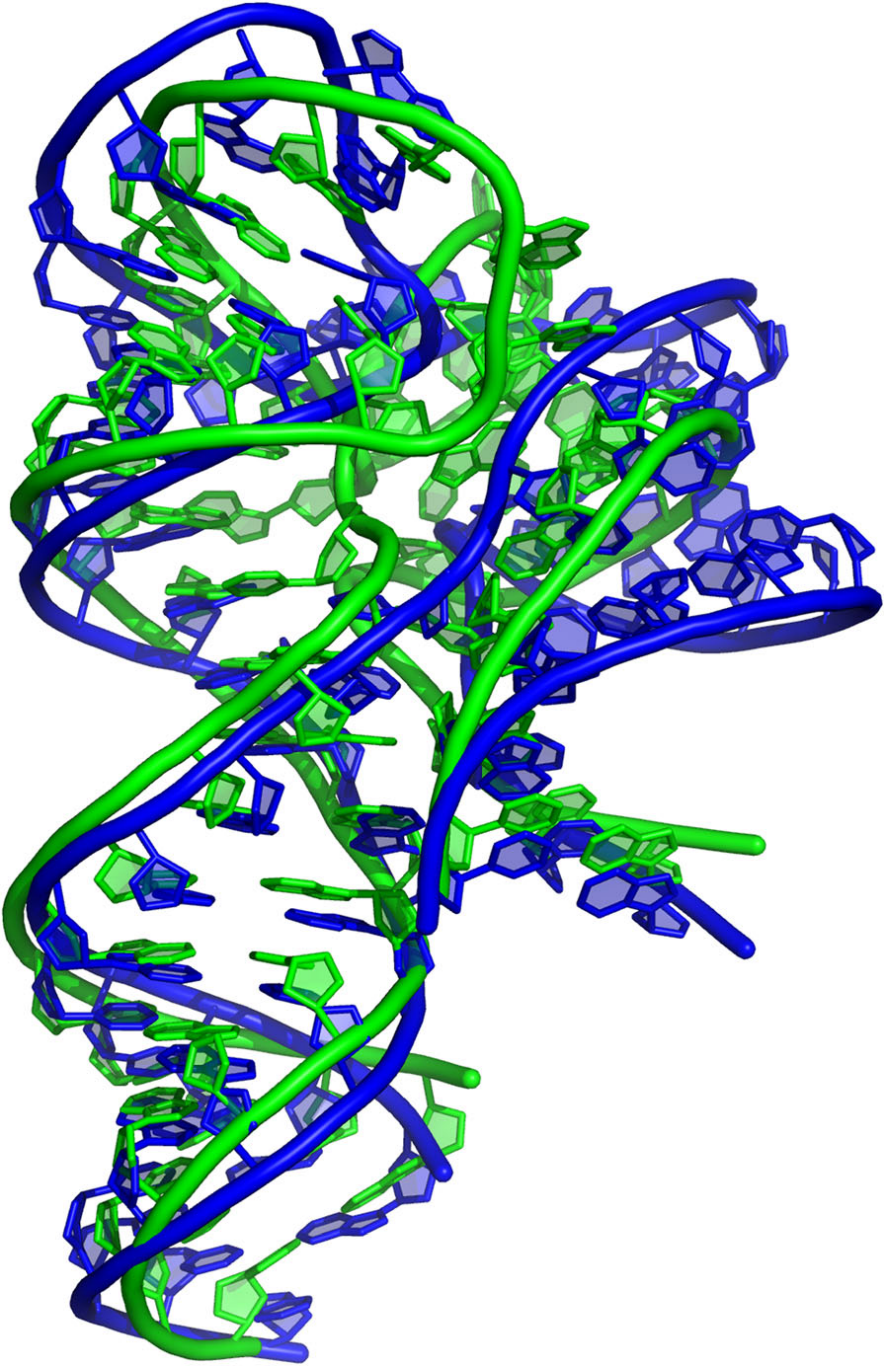
The RNA-Puzzle 13 - the ZMP riboswitch. The superposition of the native structure (green) and the EvoClustRNA prediction (blue). The RMSD between structures is 5.5 Å, the prediction was ranked as the second in the total ranking of the RNA-Puzzles (according to the RMSD values)

EvoClustRNA was tested on the RNA-Puzzle 13 problem. The target of 71 nucleotides was an RNA 5-aminoimidazole-4-carboxamide riboside 5′-monophosphate (ZMP) riboswitch, which can up-regulate de novo purine synthesis in response to increased intracellular levels of ZMP [26]. The alignment for this riboswitch was downloaded from the Rfam database (Rfam ID: RF01750), whence ten homologs were selected for modeling with Rosetta. The secondary structures for all homologs were devised with Jalview based on the Rfam alignment. The pseudoknot was suggested in the available literature [27] and it was used for modeling. The EvoClustRNA prediction with an RMSD of 5.5 Å with respect to the reference structure (Fig. 3) was the second in the total ranking of RNA-Puzzles. The final prediction was made based on the visual inspection of the best clusters, which were obtained by using the EvoClustRNA method.

**Fig. 4:**
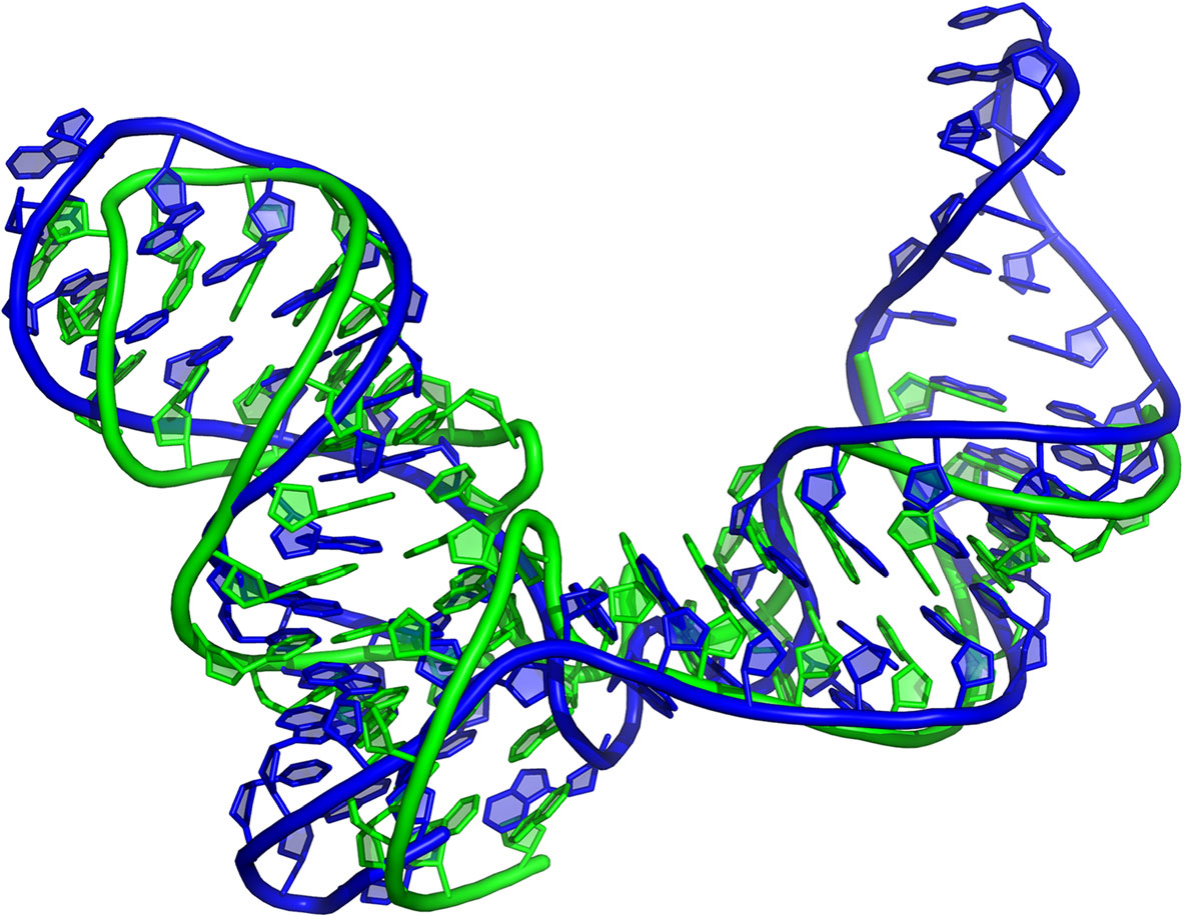
The RNA-Puzzle 14 - L-glutamine riboswitch. The RMSD between the native structure (green) and the EvoClustRNA prediction (blue) is 5.5 Å

EvoClustRNA was also used in the RNA-Puzzles for modeling problem 14. The RNA molecule of interest was the 61-nucleotide long L-glutamine riboswitch, which upon glutamine binding undergoes a major conformational change in the P3 helix [28]. It was the first RNA-Puzzle, for which the participating groups were asked to model two forms of the RNA molecule: one with a ligand (“bound”) and another one without a ligand (“free”). However, the EvoClustRNA method was used only to model the “bound” form. The alignment for this RNA family (RFAM: RF01739) was downloaded from the Rfam database, whence two homologs were selected for modeling with Rosetta. It was suggested in the literature [29] that the structure included an E-loop motif. This motif was found in the PDB database and was used as a rigid fragment during the modeling. Three independent simulations were performed and the final prediction was obtained in a fully automated manner. The native structure of the riboswitch superimposed on the model obtained with the EvoClustRNA method is shown in Fig. 4. The EvoClustRNA prediction was ranked at the first place in the overall ranking with 5.5 Å RMSD with respect to the native structure. Details of these results were reported in an article describing RNA-Puzzles Round III [10].

### Accuracy of prediction for RNA family

**Fig. 5:**
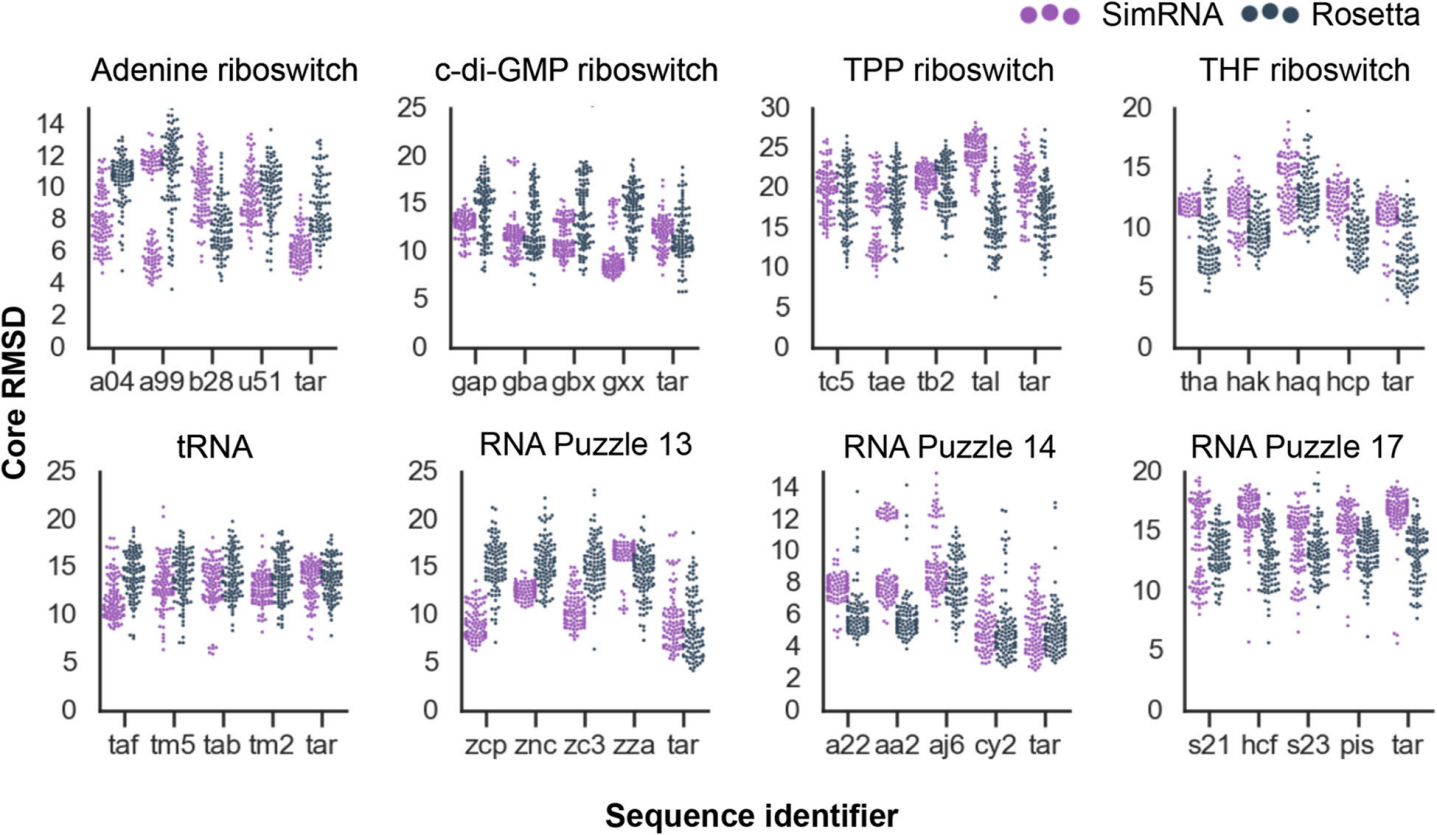
Core RMSD [Å] for the best 100 models for sequences of homologs with SimRNA and Rosetta. Tar stands for “Target” sequence. **Adenine riboswitch**: a04 (*Clostridioides difficile*, AAML04000013.1), a99 (*Streptococcus pyogenes*, AAFV01000199.1), b28 (*Oceanobacillus iheyensis*, BA000028.3), u51 (*Bacillus subtilis*, U51115.1); **c-di-GMP riboswitch**: gap (*Clostridium tetani*, AE015927.1), gba (*Bacillus halodurans*, BA000004.3), gbx (*Peptoclostridium difficile*, ABFD02000011.1), gxx (*Deinococcus radiodurans*, AE000513.1); **TPP riboswitch**: tc5 (*Xanthomonas campestris*, CP000050.1), tae (*Geobacter sulfurreducens*, AE017180.1), tb2 (*Corynebacterium diphtheriae*, BX248356.1), tal (*Streptococcus agalactiae*, AL766847.1); **THF riboswitch**: tha (*Marvinbryantia formatexigens*, ACCL02000010.1), hak (*Oribacterium sinus*, ACKX01000080.1), haq (metagenome sequence, AAQK01002704.1), hcp (*Natranaerobius thermophilus*, CP001034.1); **tRNA**: taf (*Tetrahymena thermophila*, AF396436.1), tm5 (*Rana catesbeiana*, M57527.1), tab (*Drosophila melanogaster*, AB009835.1), tm2 (*Methanothermus fervidus*, M26977.1); **RNA-Puzzle 13**: zcp (*Ralstonia pickettii*, CP001644.1), znc (*Bradyrhizobium* sp. ORS 278*,* CU234118.1), zc3 (*Ralstonia solanacearum,* CP025741.1), zza (*Caulobacter* sp. K31, CP000927.1); **RNA-Puzzle 14**: a22 (marine metagenome, AACY022736085.1), aa2 (*Synechococcus* sp. JA-2-3B’a(2–13), AACY020096225.1), aj6 (*Cyanophage phage*, AJ630128.1), cy2 (marine metagenome, AACY023015051.1) **RNA-Puzzle 17**: sequences were obtained from the alignment provided by [30]: s21 (2236876011_199011), hcf (HCF12C_58327), s23 (2210131864), pis (sequence experimentally investigated in [30])

To compare the accuracy of predictions for sequences of homologs, the core RMSD was used. The predictions were made for diverse homologous molecules that differed in sequence and length, therefore standard RMSD could not be used. Core RMSD took into account only C3′ atoms of conserved cores. The conserved cores determined based on input alignments were of the same sequence length, so there is always the same number of atoms to be compared (see Methods for details). For each RNA family, one target sequence (sequence of the reference structure taken from the PDB database) and four sequences of homologs were processed. Full names of the sequences and secondary structures used for modeling can be found in the Additional file 4, in the text and the figure, sequences will be referred to with three-letter identifiers. For different sequences that belong to the same Rfam family, divergent prediction accuracy was observed both for SimRNA and Rosetta (Fig. 5, raw data can be found in Additional file 6).

Interestingly, for 5 out of 8 RNA families for Rosetta and 4 for SimRNA, sequences of homologs yielded more accurate models than folding the target sequence. For example, in the case of the tRNA family, the best models from SimRNA were generated for a tRNA-Lys sequence (accession number: AB009835.1, referred as “tab”) from *Drosophila melanogaster* (fruit fly). These models reached a core RMSD of 5 Å, in contrast, the best model of the target sequence achieved a core RMSD of 7 Å to the reference structure. Similarly, for the TPP riboswitch, the best models from Rosetta were obtained by folding a sequence from *Streptococcus agalactiae* (AL766847.1, “tal”).

Surprisingly, SimRNA and Rosetta performed differently for the same sequences. In 26 out of 40 folded sequences, Rosetta outperformed SimRNA (models with the lowest core RMSD to the reference structure). For example, for the target sequence and all sequences of homologs of the THF riboswitch, Rosetta generated more accurate models than SimRNA. Similarly for the RNA-Puzzle 14, Rosetta in the best 100 generated more accurate models for a sequence from the marine metagenome (AACY023015051.1, “cy2“) homolog. In contrast, in the case of the adenine riboswitch, SimRNA generated more accurate models for the target sequence and a sequence from *Clostridium difficile* (AAFV01000199.1, “a99”).

Together, these data indicated that folding sequences of homologs could potentially enrich with accurate predictions a pool of models taken for clustering.

### Using MSA information to enhance the accuracy of predictions

**Fig. 6:**
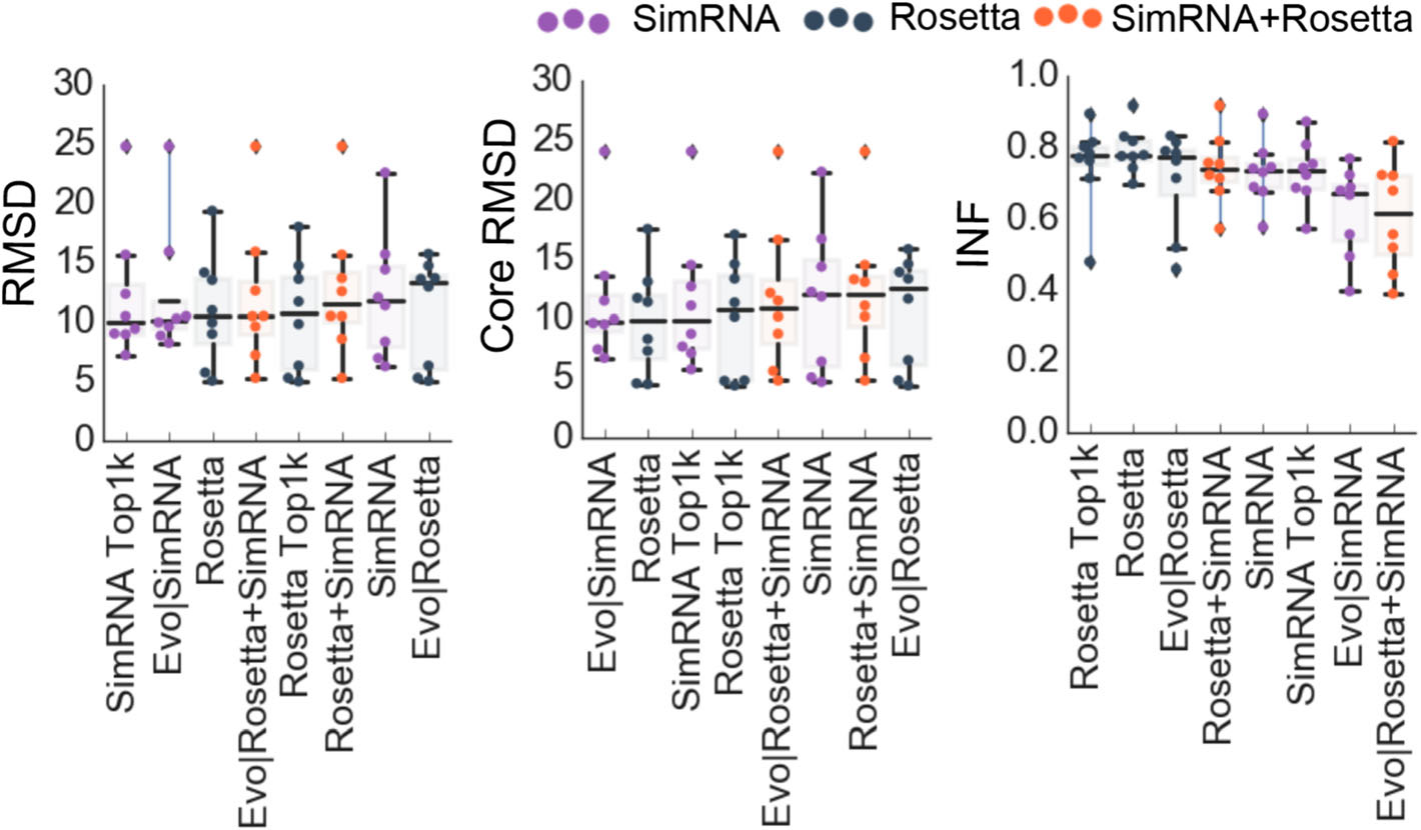
Comparison of RMSD [Å], core RMSD [Å], and INF for variants of EvoClustRNA and controls. The boxplots are sorted according to the median. For each RNA family one point - the medoid (model with the highest number of neighbors) of the biggest (first) cluster - is shown per method

To test if accurate predictions of sequences of homologs could improve the prediction of the structure of the target sequence, other variants of the method were compared to the controls, and the results are shown in Fig. 6 and the summary of the results can be found in the Additional file 5 and raw data in the Additional file 7.

The following eight variants of EvoClustRNA and controls were compared to each other. As controls, the standard protocols for Rosetta FARFAR (“Rosetta”) and SimRNA (“SimRNA”) were used. To test the clustering procedure itself without the use of any homologous sequences, three different procedures were considered where the input was: the top 500 models from SimRNA and Rosetta combined (“SimRNA+Rosetta”), the top 1000 models from Rosetta (“Rosetta Top1k”), the top 1000 models from SimRNA (“SimRNA Top1k”). The full EvoClustRNA procedure was tested with the input including 1000 models generated for five homologous sequences (the top 200 models per sequence) from SimRNA (“EvoClustRNA|SimRNA”) and Rosetta (“EvoClustRNA|Rosetta”) separately, and where 500 models (the top 100 per one sequence) produced with Rosetta and 500 models (100 per one sequence) and with SimRNA were combined into one input (“EvoClustRNA|Rosetta+SimRNA”).

SimRNA Top1k reached the lowest median of RMSD, better by 1.77 Å to control, SimRNA, and better than Evo|SimRNA by 1.61 Å. For Rosetta, Rosetta Top1k and Evo|Rosetta scored worse than the control by 0.31 Å and 2.83 Å respectively. Evo|SimRNA achieved the lowest core RMSD with the difference to the control, SimRNA, of 2.26 Å. For variants of Rosetta, the best one was the control, Rosetta. In terms of INFs, the accuracy of prediction for Rosetta and Evo|Rosetta was the same (0.77). In the case of the SimRNA, Evo|SimRNA achieved INF of 0.67 and SimRNA 0.74. The differences between benchmarked variants were not statistically significant (the Wilcoxon, non-parametric statistical test to examine if related paired samples come from the same distribution).

The comparison of the two clustering modes, half and 1-of-6 mode, can be found in the Additional file 1: Figure S1.

The analysis was performed also for various combinations of sequences of homologs (See the Additional file 2), e.g., taking the target sequence and one sequence of homolog one by one, then sequences of two homologs, then three and four in all possible combinations (Additional file 1: Figure S1). The results of an analysis of core RMSD of all possible combinations of five input sequences of homologs for all 8 RNA families investigated in this work: Adenine riboswitch (Ade), c-di-GMP riboswitch (GMP), TPP riboswitch (TPP), THF riboswitch (THF), tRNA, RNA-Puzzle 13 (RP13), RNA-Puzzle 14 (RP14), RNA-Puzzle 17 (RP17). This analysis was performed with the evox_all_variants.py from the EvoClustRNA package. Also in these tests, the statistically significant overall improvement of the prediction of variants of EvoClustRNA over the controls was not detected.

### Accurate predictions of structures for sequences of homologs

**Fig. 7:**
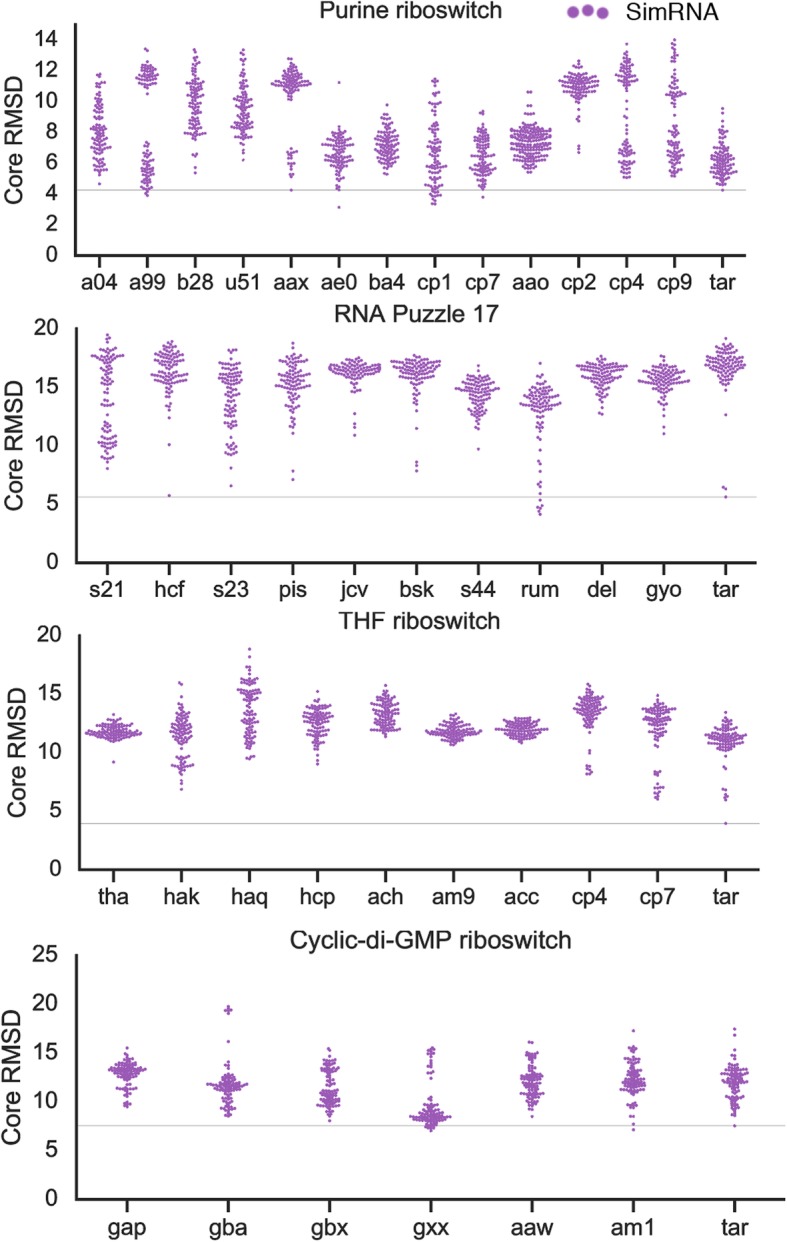
Core RMSD [Å] for the best 100 models for an extended set of sequences of homologs modeled with SimRNA (Purine riboswitch, RNA-Puzzle 17, THF riboswitch, cyclic-di-GMP riboswitch). Tar stands for “Target” sequence. The first four sequences are the same as in Fig. 5. used here for comparison to sequences of additional homologs. Full list of sequences and secondary structures used for modeling can be found in the Additional file 4. The horizontal line depicts the RMSD of the best model for the target sequence

Encouraged by the results from the folding sequences of homologs, we searched for more sequences to investigate how they fold. Because of the computational cost of predictions, we limited our analysis to four RNA families modeled with SimRNA: purine riboswitch, RNA-Puzzle 17, cyclic-di-GMP riboswitch, THF riboswitch (Fig. 7, raw data can be found in Additional file 8).

Once again, we were able to identify sequences that yielded more accurate models than the target sequence, defined as a number of models of lower core RMSD than the best model for the target. For the adenine riboswitch four sequences gave more accurate solutions, from *Streptococcus pyogenes* (AAFV01000199.1, “a99”, three models), *Bacillus cereus* (AE016877.1, “ae0”, one model), *Clostridium botulinum* (CP001581.1, “cp1”, twelve models), *Bacillus cytotoxicus* (CP000764.1 “cp07”, one model) than models for the target sequence. The best model for the “ae0” sequence was of core RMSD 3.13, which is better by 1.12 Å than the best model for target sequence (core RMSD of 4.25 Å).

In the case of the RNA-Puzzle 17, the majority of the models are close to the 20 Å, however, some homologs gave single accurate models, below core RMSD 10 Å: “hcf” (HCF12C_58327, one model), “bsk” (BS_KBB_SWE26_205m_c1114943, three models), “s23” (2236876006_041573, eleven models) (sequences and accession codes are taken from [30]). The striking case is the “rum” (RUMENNODE_3955907_1) homolog. This sequence yielded six models more accurate than the best model for the target sequence. The best of these models with the core RMSD as low as 4.13 Å was better by 1.48 Å than the best model for target sequence (core RMSD of 5.61 Å).

For the THF riboswitch, none of the sequences of homologs gave better predictions than the target sequence. Interestingly, for one of the homologs, *Alkaliphilus metalliredigens* (CP000724.1, “cp7”), a cluster of accurate solutions were generated (around 6 Å). This cluster enriched the final pool of models used for clustering and improved the selection of the final model.

In the case of the cyclic-di-GMP riboswitch, the results were consistent and comparable to the models for the target sequences and all sequences gave models of the same accuracy, with core RMSD ranging from 6.5 Å to 15 Å, after removing outliers for *Peptoclostridium difficile* (ABFD02000011.1, “gba”) sequence. Two homologs generated better models than the target sequence: AE000513.1 (“gxx”, 6 models) and AM180355.1 (“am1”, one model).

We also wanted to test if the results for sequences of homologous RNAs are consistent between simulations with different initial seed values. Seed values are numbers that are used to create initial starting points for a simulation, and are typically assigned by a pseudo random number generator. Because of the high computational cost of simulations, this analysis was done only for five cases (three independent runs with pseudo random seed values) of RNA-Puzzle 17 using SimRNA (See Additional file 9: Figure S3). The core RMSDs are not the same between runs because of the random seed values, however, the trend for some sequences (e.g., “rum”) to generate accurate models is preserved. Simulations for “JCV” sequence did not give any models below 10 Å threshold, while for “rum” sequence twenty-one models were obtained below this threshold.

### Example: sampling of conformational space for the RNA-Puzzle 17 and the TPP riboswitch

To understand whether there were structures that shared the same 3D structure in comparison with the native structure in the pool of 500 models of homologs, the results of clustering were visualized with CLANS [31]. To perform this analysis, we implemented a new tool called Clanstix (a part of the rna-tools package (https://rna-tools.readthedocs.io/en/latest/tools.html#module-rna_tools.tools.clanstix.rna_clanstix). CLANS uses a version of the Fruchterman–Reingold graph layout algorithm to visualize pairwise sequence similarities in either two-dimensional or three-dimensional space. The program was designed to calculate pairwise attraction values to compare protein sequences; however, it is possible to load a matrix of precomputed attraction values and thereby display any kind of data based on pairwise interactions. Therefore, the Clanstix program from the rna-tools package was used to convert the all-vs-all RMSD distance matrix, between selected for clustering fragments from the EvoClustRNA|SimRNAweb runs, into an input file for CLANS.

**Fig. 8:**
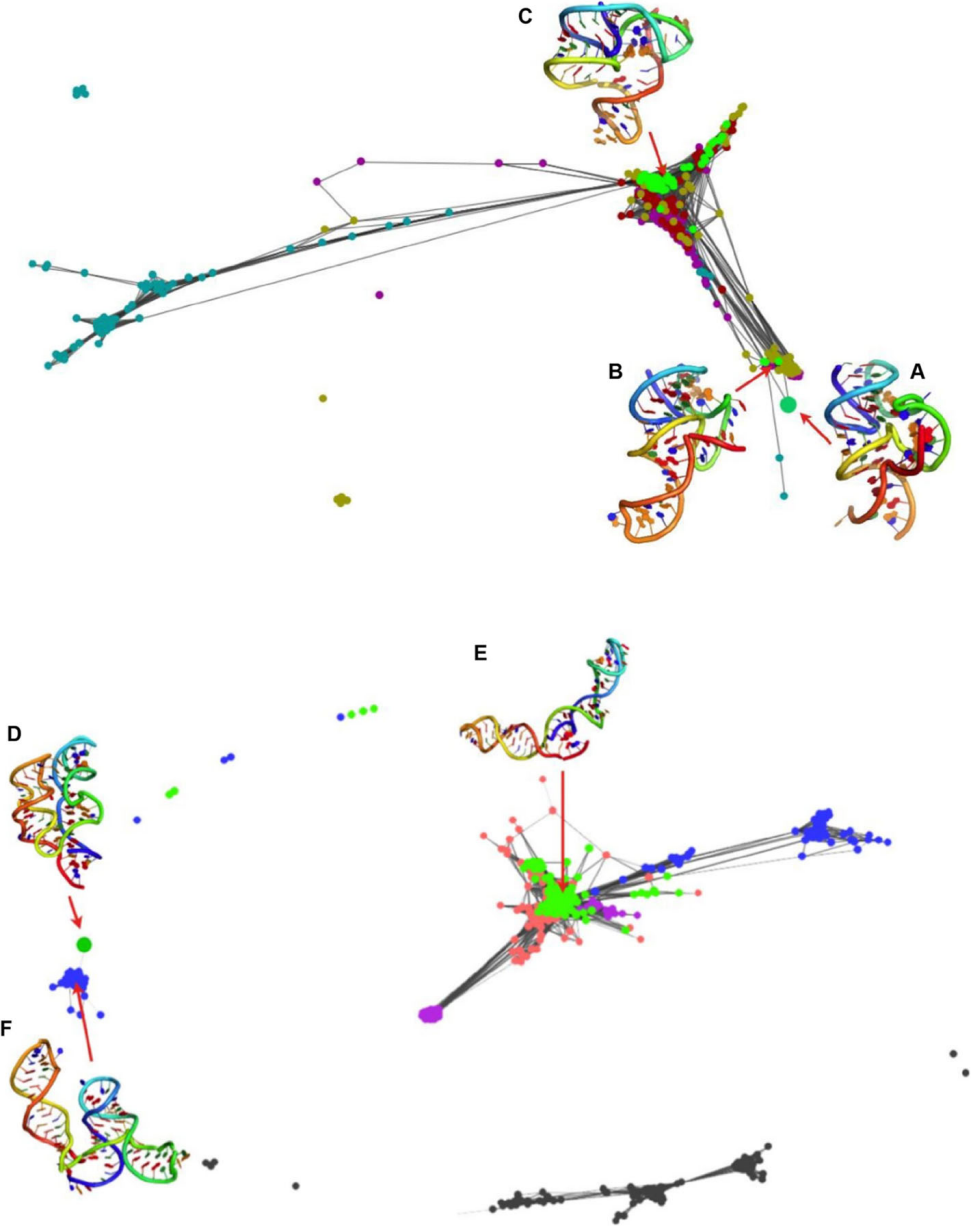
Clustering visualized with Clanstix/CLANS for RNA-Puzzle 17 and TPP riboswitch for models generated with SimRNA. RNA-Puzzle 17 (**a**-**c**): (**a**) the native structure, (**b**) the model with the close fold to the native, detected in a small cluster, (**c**) the biggest cluster with the model that was selected as the final prediction by EvoClustRNA. TPP riboswitch (**d**-**f**): (**d**) the native structure, (**e**) the model with the close fold to the native (**f**) the biggest cluster with the model that was selected as the final prediction by EvoClustRNA

The results of clustering with CLANS are shown in Fig. 8. In this clustering visualization, 100 models of five homologs are shown (each homolog uniquely colored, models of the target sequence are colored in lime). Models with a pairwise distance in terms of RMSDs lower than 6 Å are connected. The experimentally determined reference structure (Fig. 8a) was added to this clustering to see where it would be mapped. Interestingly, the native structure was mapped to a small cluster, in which there are three models for the target sequence. The cluster medoid (Fig. 8b) achieved an RMSD of 7 Å to the reference structure. This clustering visualization showed that there were models generated with the correct fold, but none of them were selected as the final prediction. In the absence of the information about the reference structure, the default prediction of EvoClustRNA was the medoid of the biggest cluster (Fig. 8c).

An analogous analysis was performed for the results of clustering of EvoClustRNA|SimRNAweb runs for the TPP riboswitch. Models with a pairwise distance in terms of RMSDs lower than 9 Å are connected. Interestingly, the reference structure (Fig. 8d, dot) was mapped to a cluster of models of one of the homologs (Fig. 8f, blue). The medoid of this cluster (Fig. 8f) achieved a core RMSD of 9 Å to the native structure. This cluster was devoid of models for the target sequence and included only models of its homologs. Since SimRNAweb was not able to detect non-canonical interactions, most of the structures were in “open” conformation and were dissimilar to the reference structure. The default prediction of EvoClustRNA (Fig. 8e) achieved an RMSD of 24 Å with respect to the reference structure.

We also looked at the diversity of models generated by the two methods used in this study. Figure 5 shows that the top 100 models from SimRNA tend to be more similar to each other as compared to the top 100 models from Rosetta. The results of clustering for the TPP riboswitch are shown in the Additional file 3. For this visualization, the top 100 models from each method were considered. The different diversity of models from each modeling method can be detected. The top 100 models generated with Rosetta were more diverse and sampled much bigger conformational space. In contrast, the top 100 models from SimRNA were similar to each other and sampled limited conformational space. This observation is important for further analysis when one combines models from different predictive methods to use them with EvoClustRNA.

## Discussion

We present a computational workflow for processing RNA alignments to perform concurrent simulations with SimRNA and Rosetta that could improve RNA 3D structure prediction. We wanted to understand if by enriching a pool of models used for clustering with models obtained from folding sequences of homologs, we can influence the selection of the final model and thus improve RNA 3D structure prediction. To test this idea, the EvoClustRNA program was implemented. The workflow is free to use and can be downloaded from https://github.com/mmagnus/EvoClustRNA.

Initially, the EvoClustRNA approach was tested on two blind RNA-Puzzles challenges. The predictions ranked as the second for the ZMP riboswitch (RNA-Puzzle 13) as the first of all submissions for the L-glutamine riboswitch (RNA-Puzzle 14). Encouraged by these results, we tested the method on a dataset of 8 RNA families.

The clustering results shown in Fig. 8. shows that EvoClustRNA was able to sample conformational space efficiently and near-native structures were generated during simulations. Incorrect predictions were made because of the problem with the energy function to score models properly and the accurate models were not enriched in the top 100. This kind of visualization could prompt new hypotheses to be tested experimentally, in contrast with folding a single sequence only.

We discovered several cases in which sequences of individual homologs were folded to more accurate structures than the original target sequence. This observation demonstrated that RNA 3D structure prediction can be improved by the consideration of sequences homologous to the target sequence. However, many other homologs folded poorly and were not helpful. Further investigation may allow sequence features to be identified that would allow better curation of sequences of homologs that are more likely to lead to convergent models. Interestingly, the computational “foldability” of a sequence depends on which package is used, SimRNA or Rosetta (Fig. 5), perhaps relating to different libraries of fragments that the different packages use, or different choices in modeling helices, particularly pseudoknots. Another potential solution would be to investigate if this “foldability” is related to free energy calculated by secondary structure prediction methods or to the potential of particular sequence variants to form stable structures and crystallize [4, 32, 33].

The workflow described in this study can be combined with any method for RNA tertiary structure prediction, and this is one of the possible lines of further research. As shown here, SimRNA and Rosetta achieved different prediction accuracy depending on the folded sequence, e.g., for the THF riboswitch (Fig. 5, “tha” sequence). Therefore, other RNA 3D structure prediction methods could be tested to see if they enrich the pool of accurate models used for clustering with EvoClustRNA.

The approach described here could be combined with direct-coupling analysis, proposed for example by [14, 15]. In this approach, a DCA analysis should be performed for an alignment to generate restraints for several homologous sequences. These sequences could be then folded and EvoClustRNA could be applied to select the final model or to visualize possible folds of an RNA molecule.

## Conclusions

We present a complete bioinformatics workflow for processing RNA alignments to perform concurrent simulations with different RNA 3D structure prediction methods, here exemplified by SimRNA and Rosetta. The workflow has proven useful for RNA modeling, as revealed by successful predictions for the RNA-Puzzles experiment [10]. At the current stage, the fully-automated method does not always provide a significant improvement over single sequence modeling. However, we discovered several striking cases in which particular homologs were folded to more accurate models than the original target sequence. This work, for the first time to our knowledge, demonstrates the importance of the selection of the target sequence (from many variants in a multiple sequence alignment) for the success of RNA 3D structure prediction. This discovery prompted both Bujnicki and Das lab to use modeling of sequences of homologs in RNA-Puzzles and became a new routine in the modeling pipeline. To support new research in this area, we provide all relevant scripts in a documented and ready-to-use form. By exploring new ideas and identification of limitations of the current RNA 3D structure prediction methods, this work is bringing us closer to the near-native computational RNA 3D models.

## Material & Methods

### Reference structures

All structures solved experimentally and used in this study were obtained from the Protein Data Bank [34] and parsed to a standardized format with rna-tools (https://github.com/mmagnus/rna-tools).

### Benchmark dataset

To evaluate the performance of the presented methodology, we compiled a dataset of 8 RNA sequences: five RNA sequences from [14]: Adenine riboswitch (Ade, PDB ID: 1Y26, RFAM ID: RF00167) [35], Thiamine pyrophosphate-sensing riboswitch (TPP, PDB ID: 2GDI, RFAM ID: RF00059) [36], tRNA (PDB ID: 1FIR, RFAM: RF00005) [37], c-di-GMP-II riboswitch (cdiGMP, PDB ID: 3Q3Z, RFAM ID: RF01786) [38], Tetrahydrofolate riboswitch (THF, PDB ID: 4LVV, RFAM ID: RF00059) [39] and three RNA-Puzzles: 13 (5-aminoimidazole-4-carboxamide ribonucleotide riboswitch, ZMP riboswitch, PDB ID: 4XW7, Rfam id: RF01750) [26], 14 (L-glutamine riboswitch, GlnA, PDB ID: 5DDO, RFAM ID: RF01739) [28], 17 (Pistol ribozyme, PDB ID: 5K7C, RFAM ID: RF02679) [40].

### Multiple sequence alignment generation and selection of homologs

Each query sequence was taken from the corresponding PDB file. The MSA was obtained from the Rfam database [41] and in the case of the Pistol ribozyme, the MSA was published as the supplementary data provided by [30]. MSAs were reduced (using JalView [42], sequence similarity threshold 90%) to keep only diverse representatives. In theory, all sequences could be folded but because of the computational costs of simulations (6-10 h per sequence for 80 CPUs, using either SimRNAweb or Rosetta FARFAR), we decided to fold only four of the shortest sequences from the MSA. Once the final set of homologs to be folded was selected, the positions common to all sequences selected were determined.

The list of all the sequences and secondary structures used in the benchmark of EvoClustRNA and a list of links to the SimRNAweb predictions can be found in Additional file 4.

### RNA 3D structure prediction

For each sequence chosen for folding, secondary structure predictions were generated based on the MSA. Two methods were used in this study: SimRNA and Rosetta. For Rosetta, a total of 10,000 decoys were generated for the target sequence and each homologous sequence using the Rosetta FARFAR protocol [22]. For SimRNA prediction, the SimRNAweb server was used [43] using the default parameters.

Both modeling steps can be performed in a semi-automated way with rna-tools (M.M. et al., unpublished, software available for download at https://github.com/mmagnus/rna-tools) as well as the pipeline of tools facilitating modeling with Rosetta (https://rna-tools.readthedocs.io/en/latest/tools.html#rosetta) and SimRNA/SimRNAweb (https://rna-tools.readthedocs.io/en/latest/tools.html#simrnaweb).

#### The Rosetta method

The method used to generate and select models has been described previously [44], but will be reviewed here briefly. Inspired by the Rosetta protein modeling tool [45] methodology, Fragment Assembly of RNA (FARNA) predicts the tertiary structure by assembling short 3-residue fragments, and then sampling using a Monte Carlo algorithm, guided by a knowledge-based energy function. The method was improved in 2010 by adding new energy terms within the force field specific for RNA molecules. The improved method was called Fragment Assembly of RNA with Full-Atom Refinement (FARFAR). This FARFAR protocol was used for modeling in this work. A total of 10,000 independent simulations are carried out (starting from different random number seeds) for each query sequence, and the resulting structures are clustered as previously reported [44]. For short RNA fragments (up to 32 nucleotides) Rosetta can be accessed via the “Rosetta Online Server That Includes Everyone” (ROSIE) [46]. However, in this work much longer sequences were modeled, so the Rosetta package was used locally at the HPC (High-Performance Computing) provided by the International Institute of Molecular and Cell Biology or, for the ZMP riboswitch RNA-Puzzle, on the Stanford BioX^3^ cluster.

#### The SimRNA method (as implemented in the SimRNAweb server)

SimRNAweb [43] is a user-friendly online interface for modeling RNA 3D structures using SimRNA [21]. SimRNA uses a coarse-grained representation of RNA molecules, the Monte Carlo method to sample the conformational space, and relies on a statistical potential to describe the interactions in the folding process. SimRNAweb makes SimRNA accessible to users who do not normally use high-performance computational facilities or are unfamiliar with using the command line tools. The simplest input consists of an RNA sequence to fold RNA de novo. Alternatively, a user can provide a 3D structure in the PDB format, for instance, a preliminary model built with some other technique, to jump-start the modeling close to the expected final outcome. The user can optionally provide secondary structure and distance restraints and can freeze a part of the starting 3D structure. The web server is available at http://genesilico.pl/SimRNAweb. In this work, all simulations were performed using the default parameters of the server. The lowest energy 100 and 200 models (called also in this work the top 100 and top 200) were generated based on SimRNA trajectories using rna-tools, i.e., the rna_simrnaweb_download_job.py script (https://rna-tools.readthedocs.io/en/latest/tools.html#simrnaweb).

### Selection of common positions (conserved core)

Structural fragments corresponding to the evolutionarily conserved regions (common for all homologs) determined from the alignment are processed using evoClustRNA.py resulting in an all-vs-all core RMSD matrix. Next, the matrix is passed to the clustering script, evoClust_clustix.py to perform automated clustering in two modes: “1-of-6” and “half”.

### Clustering routine

EvoClustRNA uses the clustering procedure implemented earlier by Irina Tuszyńska for the analysis of RNA-protein complex models [47] and used in the NPDock server [48]. The method is an implementation of an algorithm used for clustering with Rosetta for protein structure prediction [49], also described in [17].

Briefly, a fraction of lowest-energy structures for each homolog is taken for clustering. The clustering procedure is iterative and begins with calculating a list of neighbors for each structure. Two structures are considered as neighbors when the RMSD between them is smaller than a given distance cutoff. evoClust_clustix.py in the package is a program that performs a clustering for a user-defined cutoff, e.g., for RMSD equal to 7 Å. However, to find a proper cutoff, an iterative procedure of clustering starts from 0.5 Å and is incremented by 0.5 Å, until the required criterion is met. Two criteria were tested in this work, called “1-of-6” and “half.” In the “1-of-6” mode, the clustering was stopped when the first (the biggest) cluster contained 1/6 of all structures taken for clustering. For example, for five homologs, 500 structures were clustered and an iterative clustering stopped when the first cluster contained over 80 structures. In the second mode tested, “half,” the clustering procedure was finished when the first three clusters contained over half of the structures. Thus, for five homologs, 500 structures were clustered, and the iterative clustering stopped when there were at least 250 structures in the three biggest clusters. This iterative procedure is implemented in evoClust_autoclustix.py that is a wrapper for evoClust_clustix.py.

### Model selection

The final 3D model for the target sequence is the first occurrence of the model for the reference sequence in the clustering output starting from the top of the file. It there is no model for the reference sequence in the first cluster, then the second cluster is processed, and so on. This analysis is done by evoClust_get_models.py automatically based on the output files generated by the clustering procedure.

### Workflow implemented as EvoClustRNA

The scripts to perform the analysis are implemented in Python 3 and freely available at https://github.com/mmagnus/EvoClustRNA with the detailed documentation under the link http://evoclustrna.rtfd.io.

### Evaluation

To assess the accuracy of predictions (1) the Root Mean Square Deviation (RMSD) is used to compare models to reference structures based on the Euclidean distance between a given pair of corresponding atoms and (2) the Interaction Network Fidelity (INF) is used to compare networks of interactions (base pairing, stacking) between models and reference structures.

RMSD is defined by the following formula:

$$ RMSD=\sqrt{\frac{1}{N}\sum \limits_{i=1}^N{\delta}_i^2} $$

where δ is the Euclidean distance between a given pair of corresponding atoms. RMSD is calculated for all heavy atoms.

Secondary structure comparisons are calculated based on outputs of ClaRNA [50] using the Interaction Network Fidelity (INF) value that is computed as:

$$ INF=\sqrt{\left(\frac{TP}{TP+ FP}\right)\times \left(\frac{TP}{TP+ FN}\right)} $$

where TP is the number of correctly predicted base-base interactions, FP is the number of predicted base-base interactions with no correspondence in the solution model, and FN is the number of base-base interactions in the solution model not present in the predicted model [10].

Both metrics mentioned above, RMSD and INF, are used to calculate the distance between the generated models and reference structures. However, they cannot be applied directly to compare models for diverse homologous molecules that differ in sequence and length. So to deal with this issue, a new metric based on RMSD was implemented as core RMSD. Core RMSD considers only C3′ atoms of conserved cores (that are of the same size). The conserved cores determined based on input alignments are of the same sequence length, so there is always the same number of atoms to be compared. However, full atom RMSD for the cores cannot be calculated because the sequences can vary. That is why only a single atom, C3′, is used in this metric. Naturally, this metric is not only used for evaluation of the accuracy of predictions but also for clustering.

Calculations for evaluation of predictions are performed with evoClust_calc_rmsd.py program that is built around Biopython [51].

### Structure visualizations

Structure visualizations in 3D were generated with PyMOL (version 1.7.4 Edu Enhanced for Mac OS X by Schrödinger) [52].

### Statistical analyses

Statistical analyses and visualization of the data were carried out with Python 2.7 using following Python packages: Matplotlib [53], Pandas, Seaborn [54], Jupyter (former IPython) [55]. The differences between benchmarked variants were tested with the Wilcoxon non-parametric statistical test implemented in SciPy.

## Supplementary information

**Additional file 1: Figure S1.** The comparison of two clustering mode, "half" and "1-of-6" (related to Fig. 6).

**Additional file 2:** The analysis was performed also for various combinations of sequences of homologs (related to Fig. 6). The results of an analysis of core RMSD of all possible combinations of five input sequences of homologs for all 8 RNA families investigated in this work: Adenine riboswitch (Ade), c-di-GMP riboswitch (GMP), TPP riboswitch (TPP), THF riboswitch (THF), tRNA, RNA-Puzzle 13 (RP13), RNA-Puzzle 14 (RP14), RNA-Puzzle 17 (RP17). This analysis was performed with the evox_all_variants.py from the EvoClustRNA package. Each sequence of homologs was ordered from 1 to 3. A mode “h1” means models of the first homolog and the target sequence used for clustering, “h2” means models of the second homolog and the target sequence. “h234” means that models of three homologs were considered during clustering, the second homolog, third and fourth. For each variant 5 top clusters are shown and the first cluster is marked with a black dot. The first panel combines the results for SimRNA and Rosetta, the second panel shows the results for SimRNA and the third only for Rosetta.

**Additional file 3: Figure S2.** The comparison of top100 of Rosetta and SimRNA. Top 100 models from SimRNA vs Rosetta visualized with Clanstix/CLANS for models of the target sequence for the TPP riboswitch. Models obtained with (A) Rosetta and (B) SimRNA. Top 100 models from Rosetta are very different from each other and they cluster around the correct, reference structure (pointed by the red arrow). Top 100 models from SimRNA showed less diverge and cluster all altogether.

**Additional file 4:** List of all the sequences and secondary structures used in the benchmark of EvoClustRNA and a list of links to the SimRNAweb predictions.

**Additional file 5:** Summary of analysis for Fig. 6.

**Additional file 6:** All data required to generate Fig. 5.

**Additional file 7:** All data required to generate Fig. 6.

**Additional file 8:** All data required to generate Fig. 7.

**Additional file 9: Figure S3.** Analysis of replicates for SimRNA simulations with different initial seed values for RNA Puzzle 17.

## Data Availability

The datasets generated and/or analyzed during the current study are available in the EvoClustRNA repository, https://github.com/mmagnus/EvoClustRNA

## Abbreviations

INF: Interaction Network Fidelity
PDB: Protein Data Bank
RMSD: Root mean square deviation
