# Supplementary figures and images for: RNA 3D structure prediction guided by independent folding of homologous sequences
Source: BMC Bioinformatics. 2019 Oct 22;20:512. doi: 10.1186/s12859-019-3120-y (PMC6806525; doi:10.1186/s12859-019-3120-y)

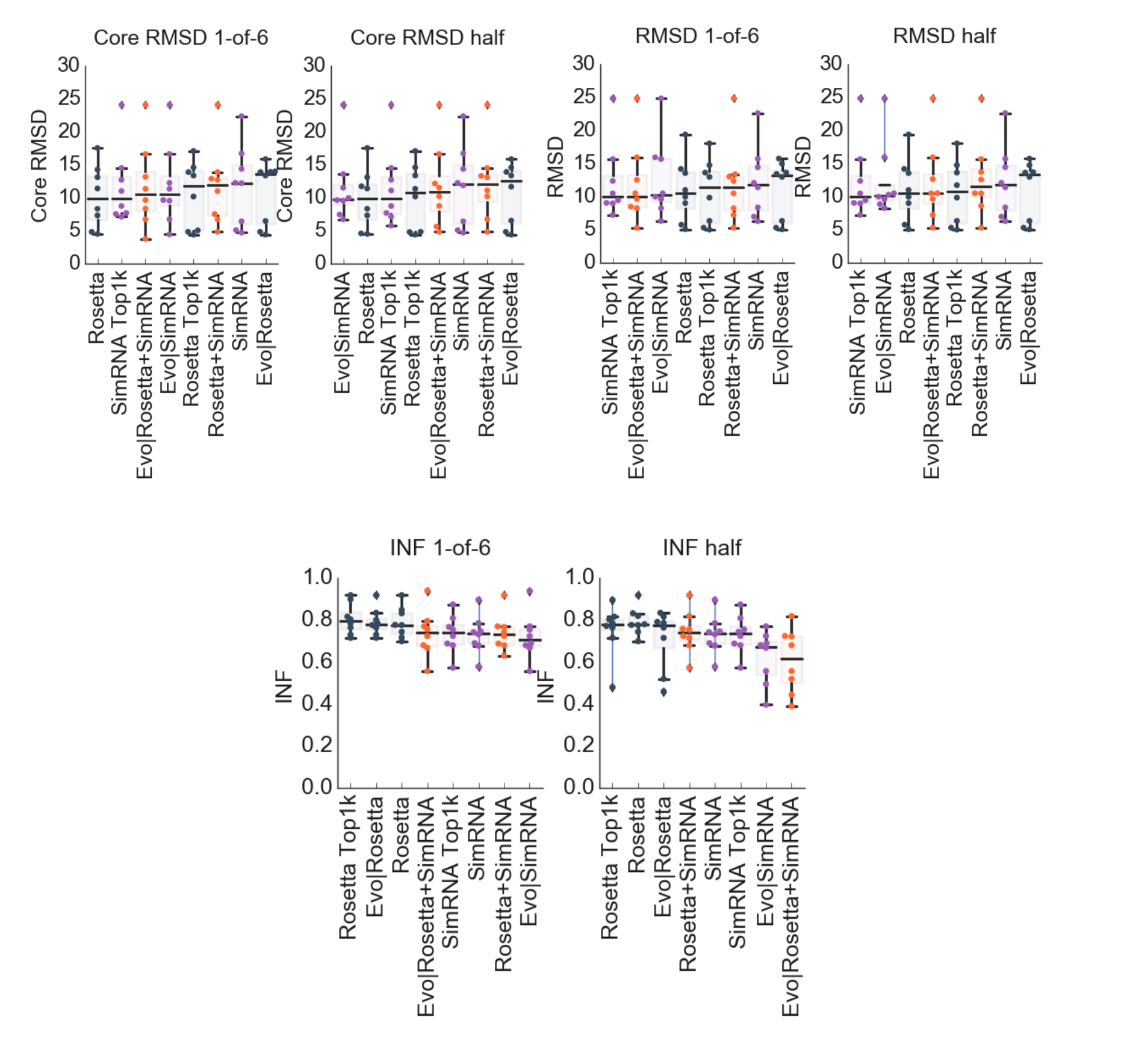

Supplement: Supplementary file 1 — Additional file 1: Figure S1. The comparison of two clustering mode, "half" and "1-of-6" (related to Fig. 6). [file 12859_2019_3120_MOESM1_ESM.png]

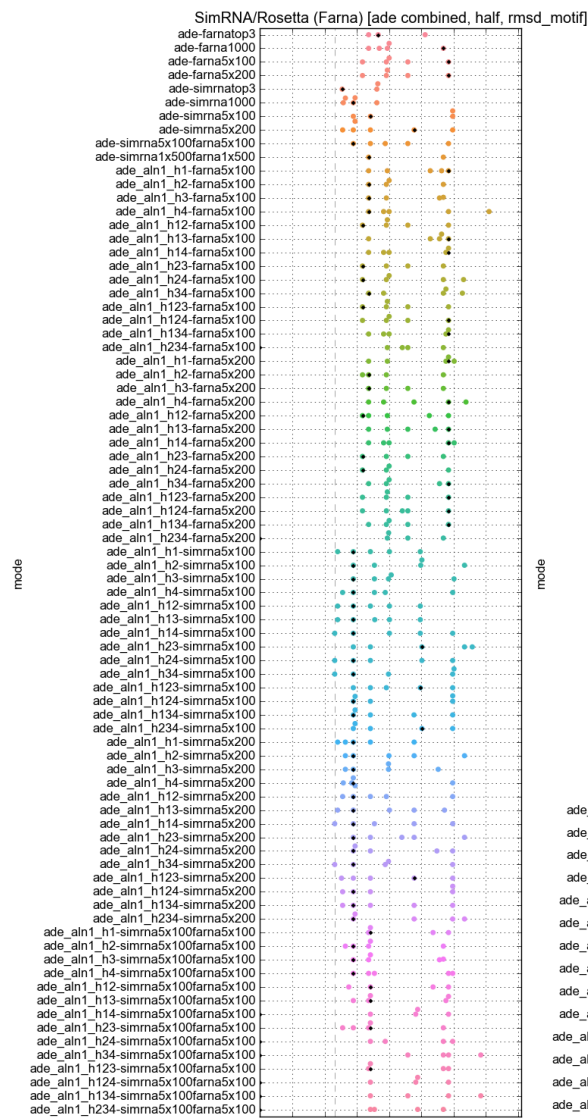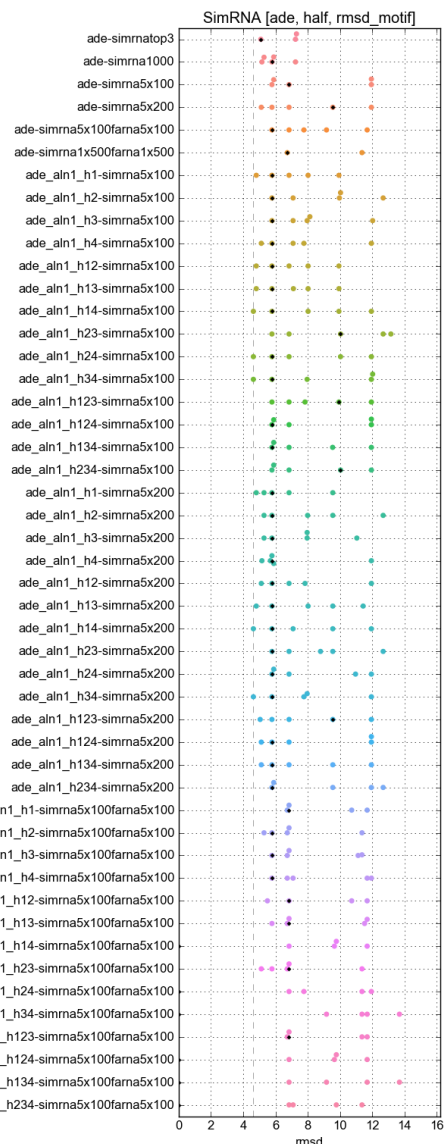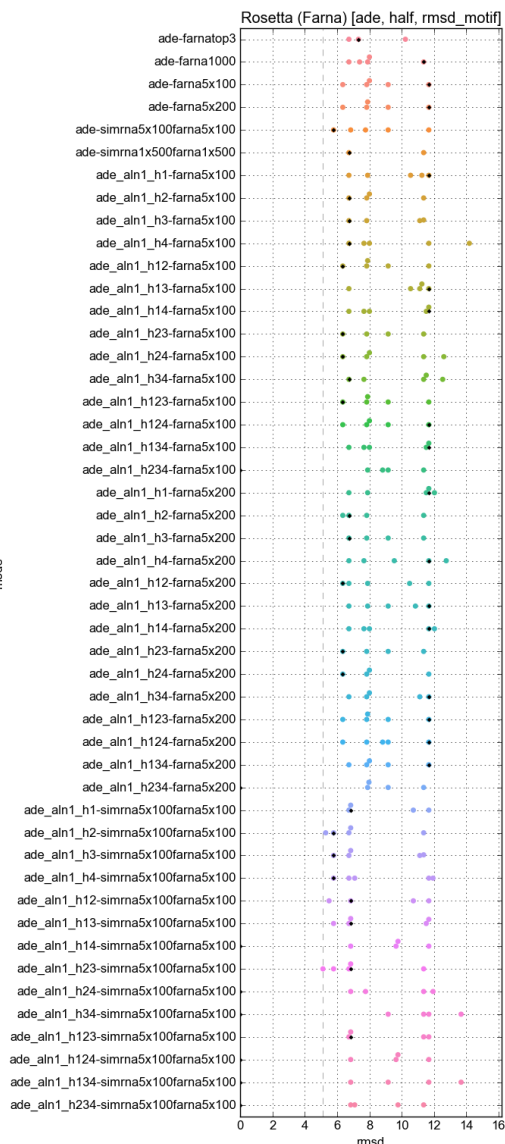

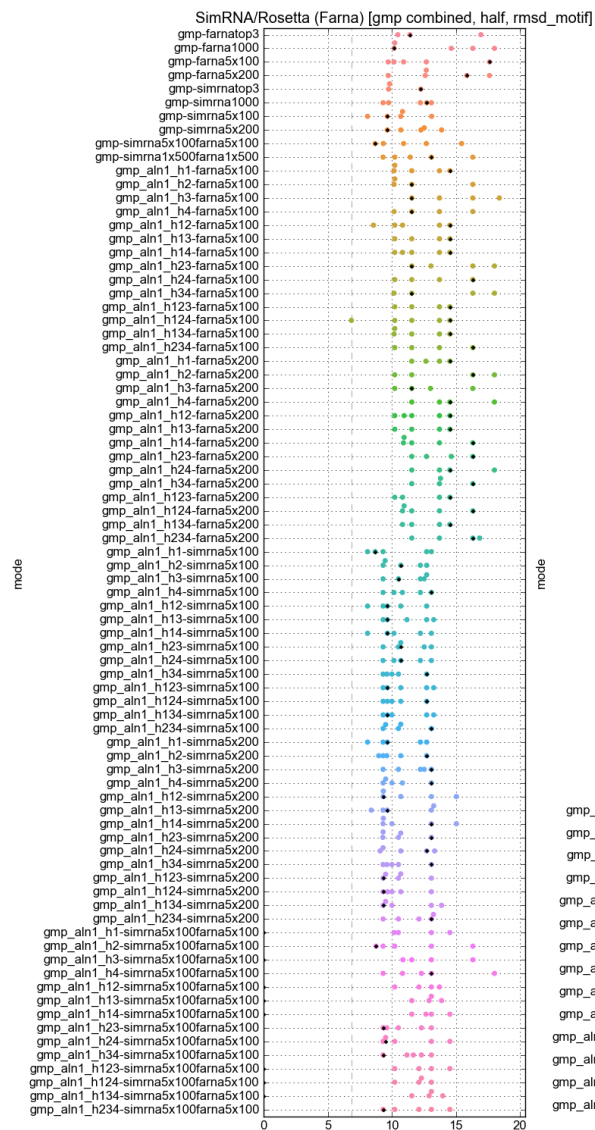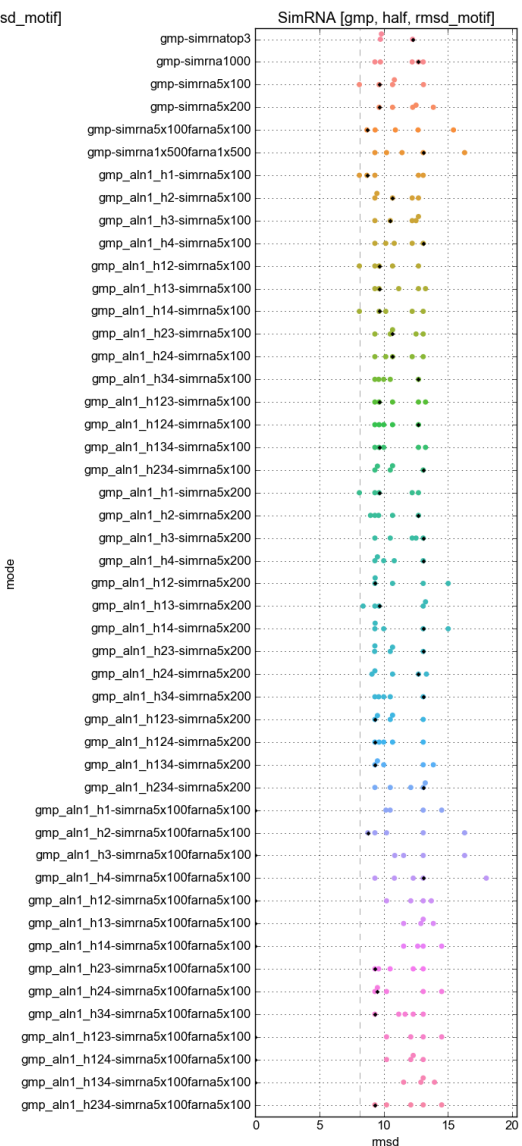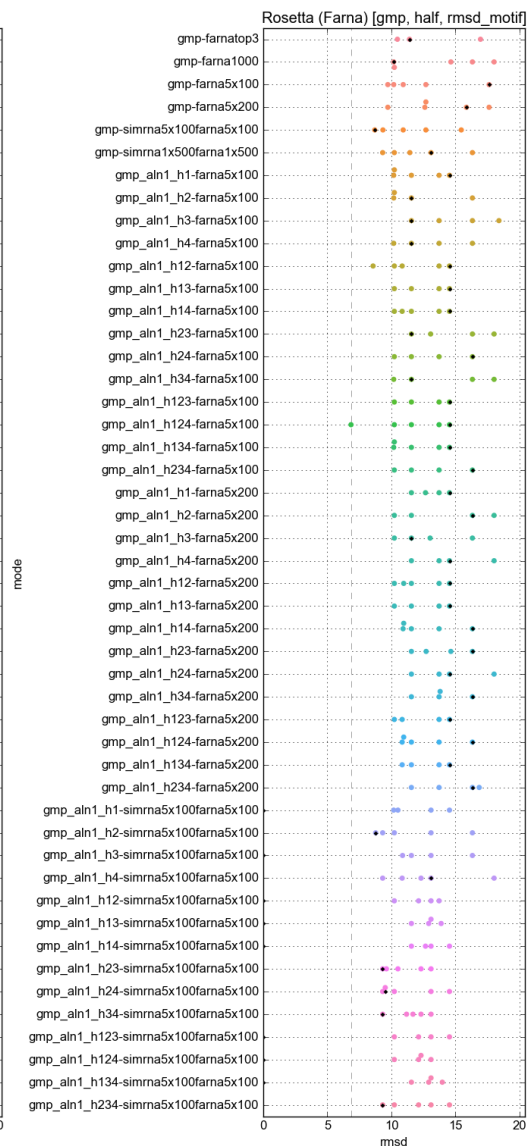

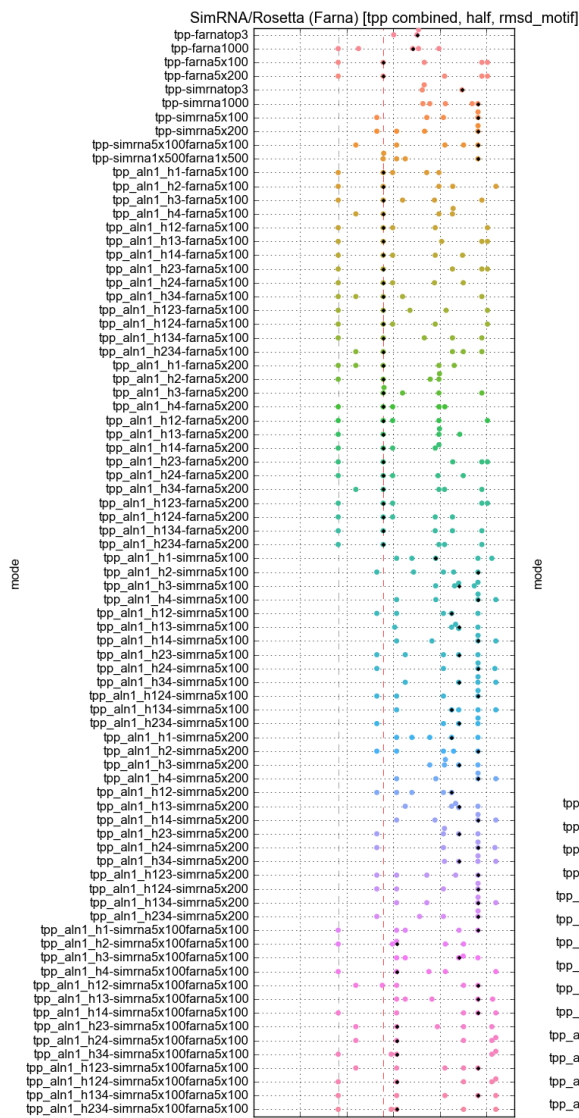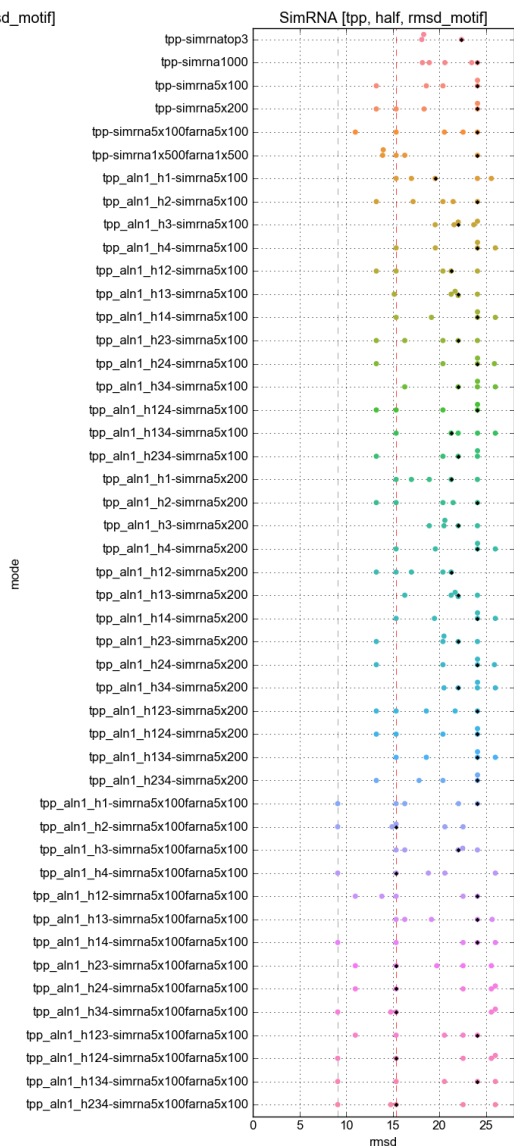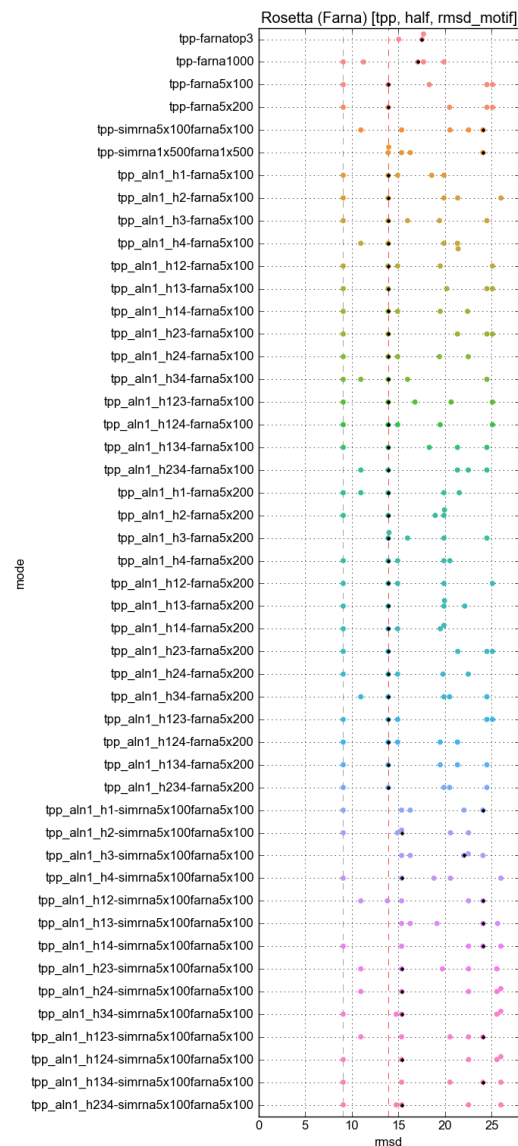

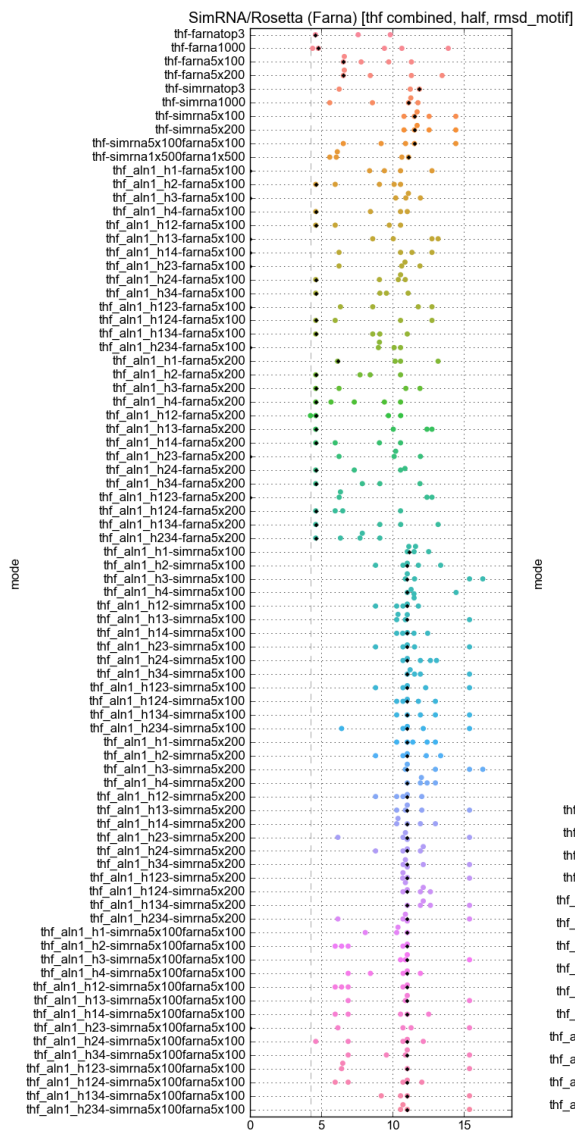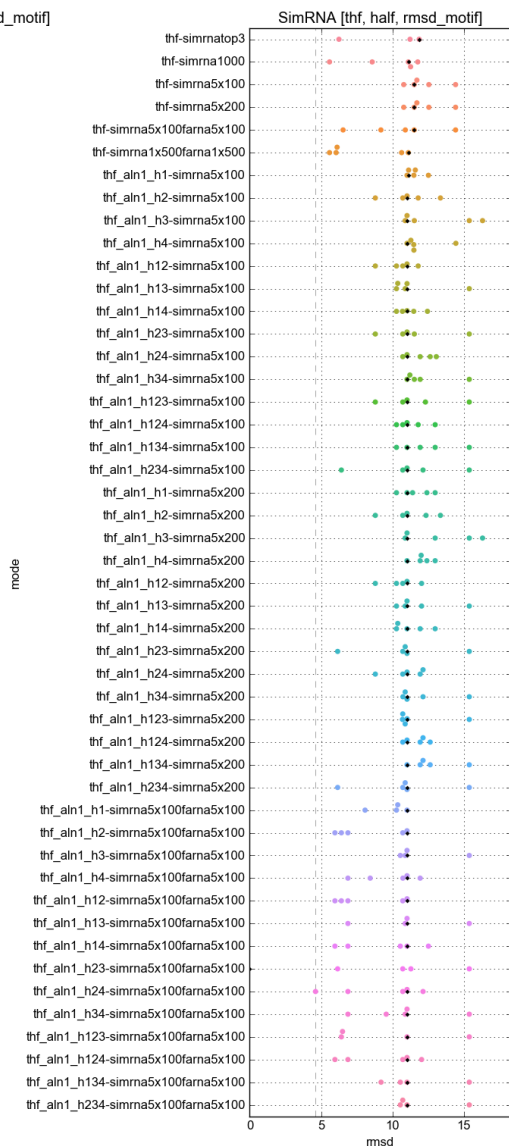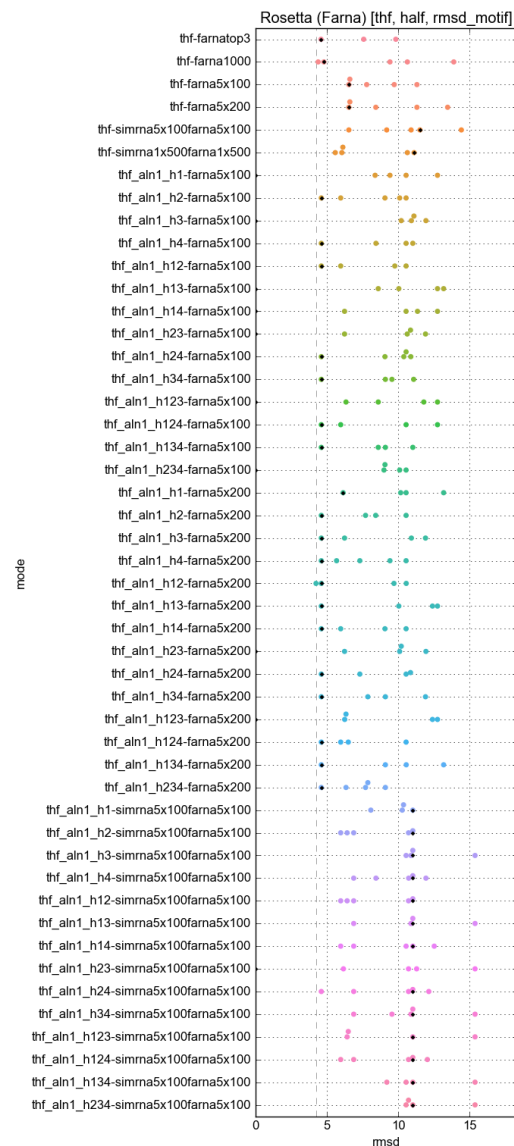

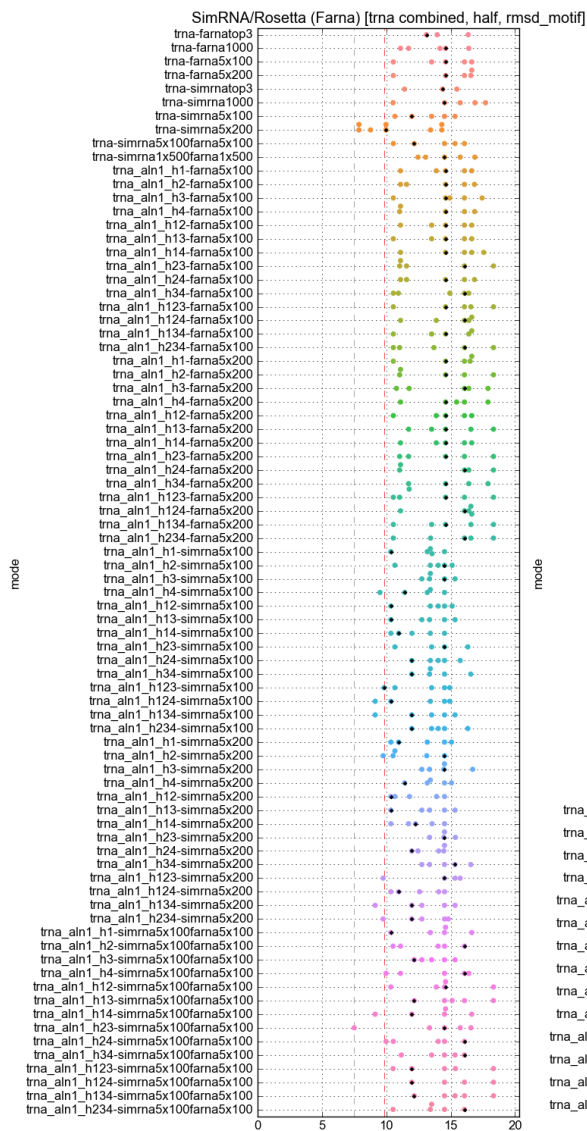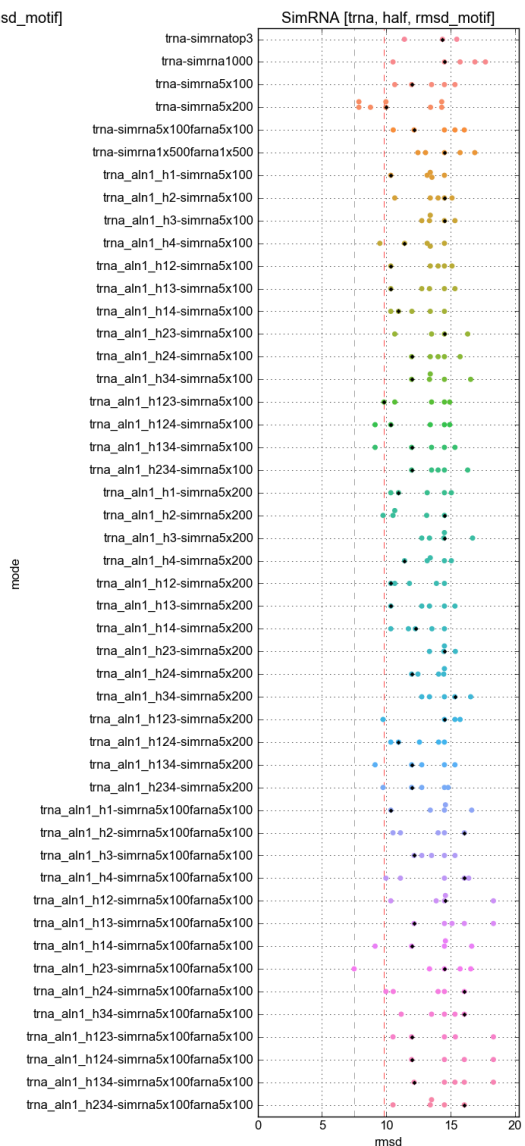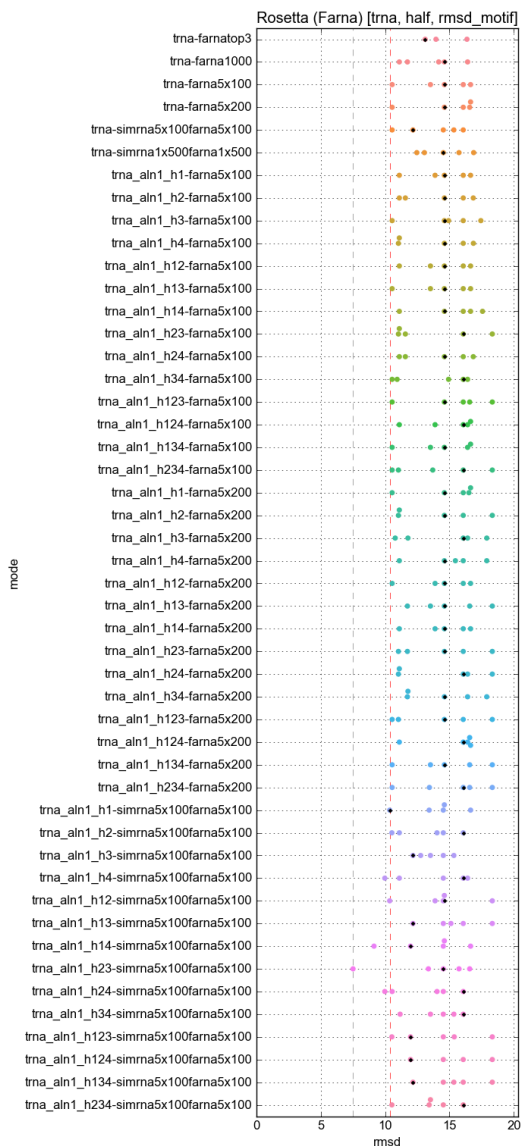

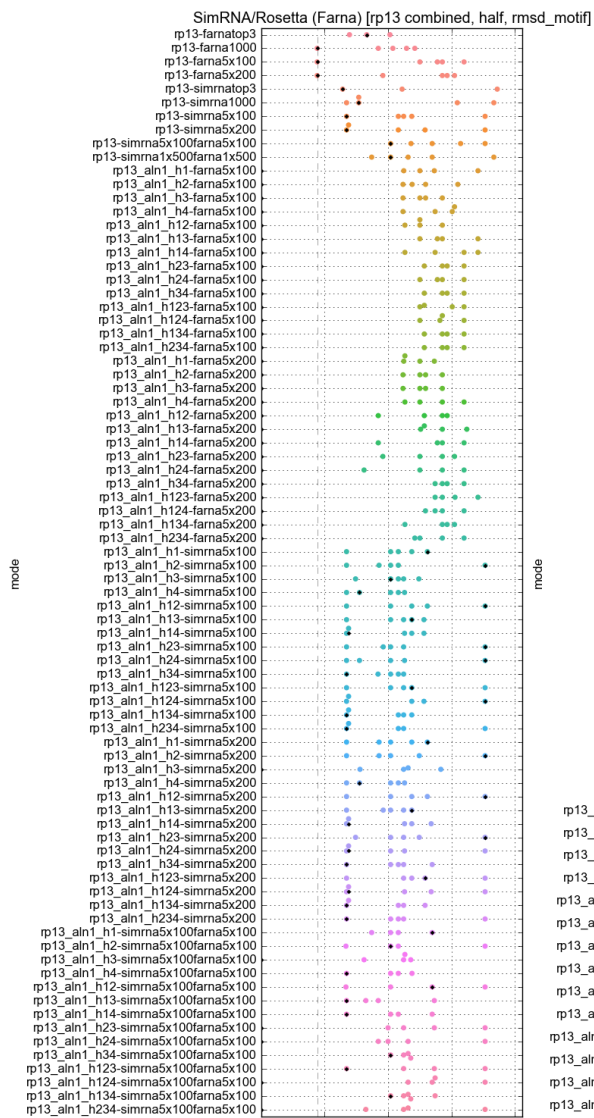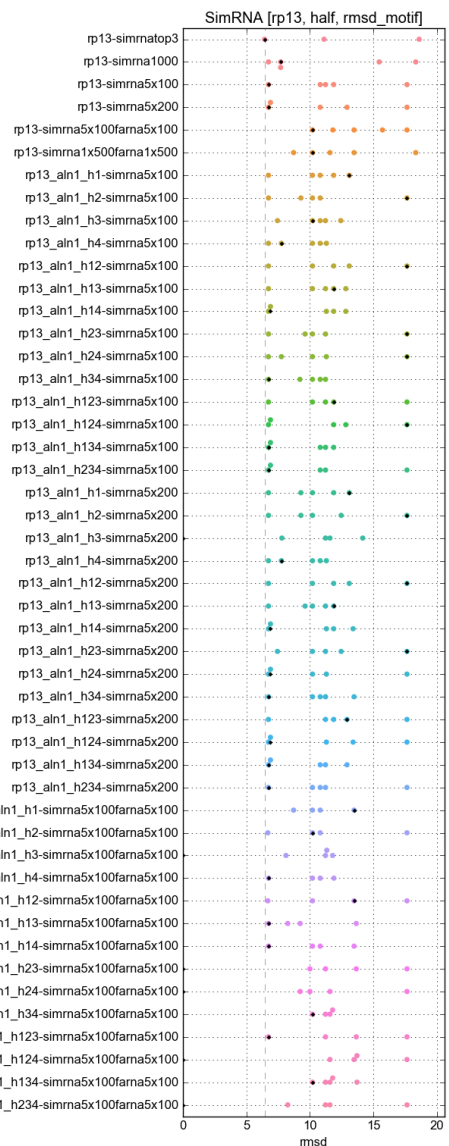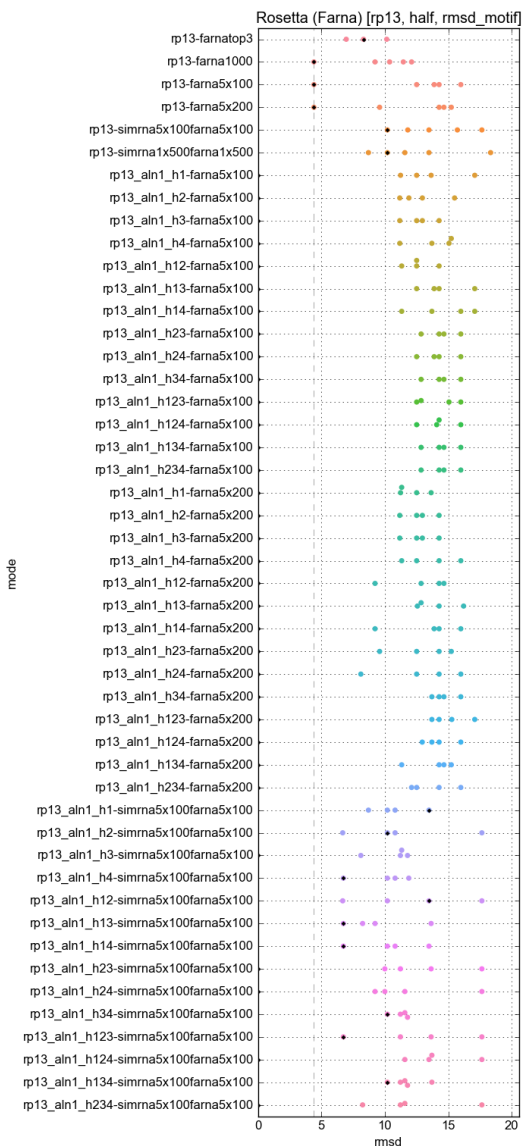

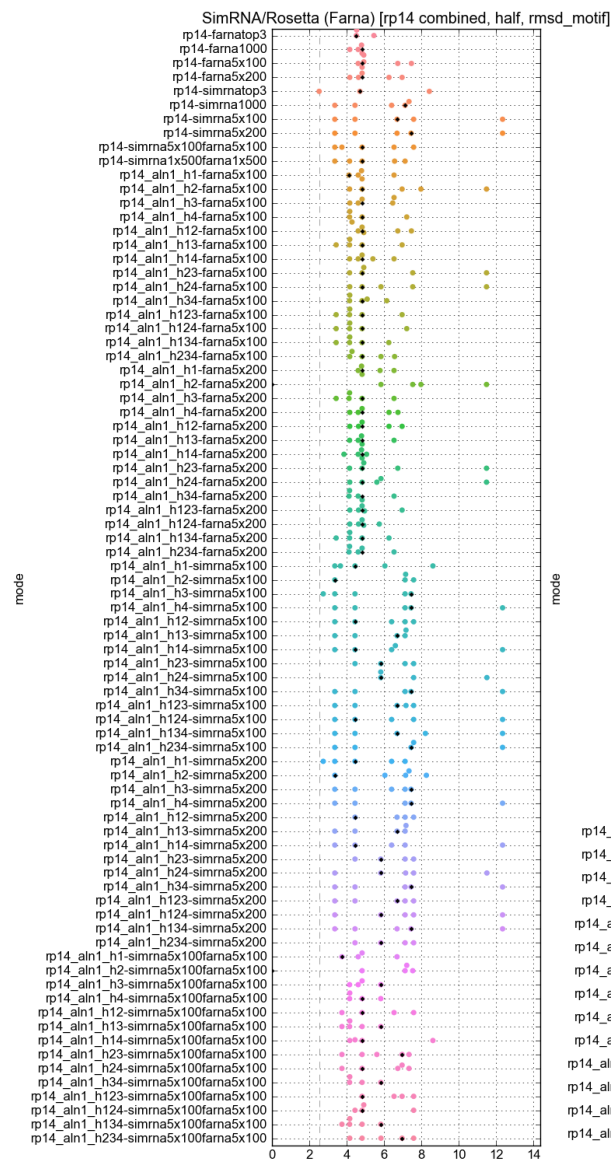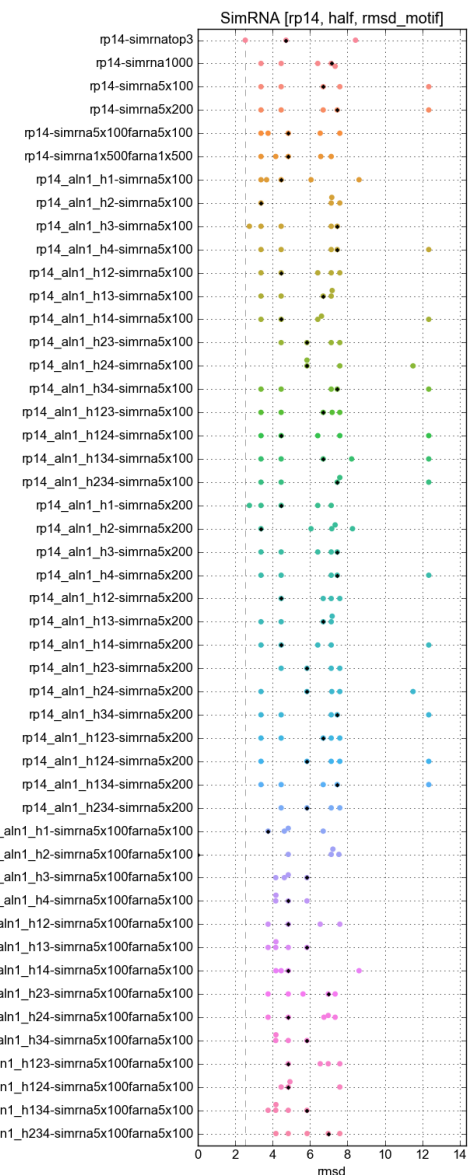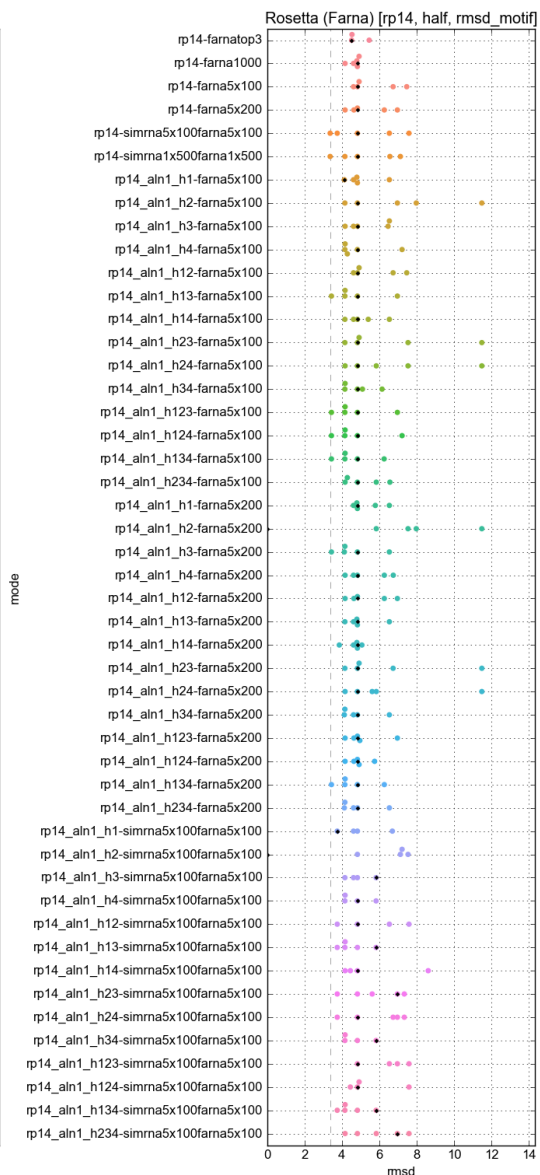

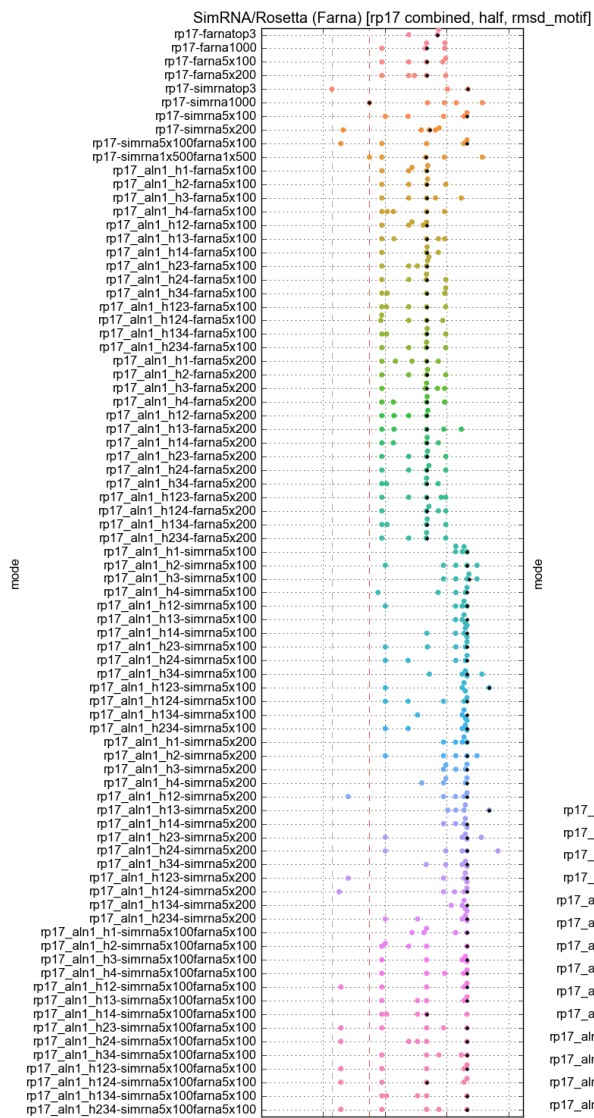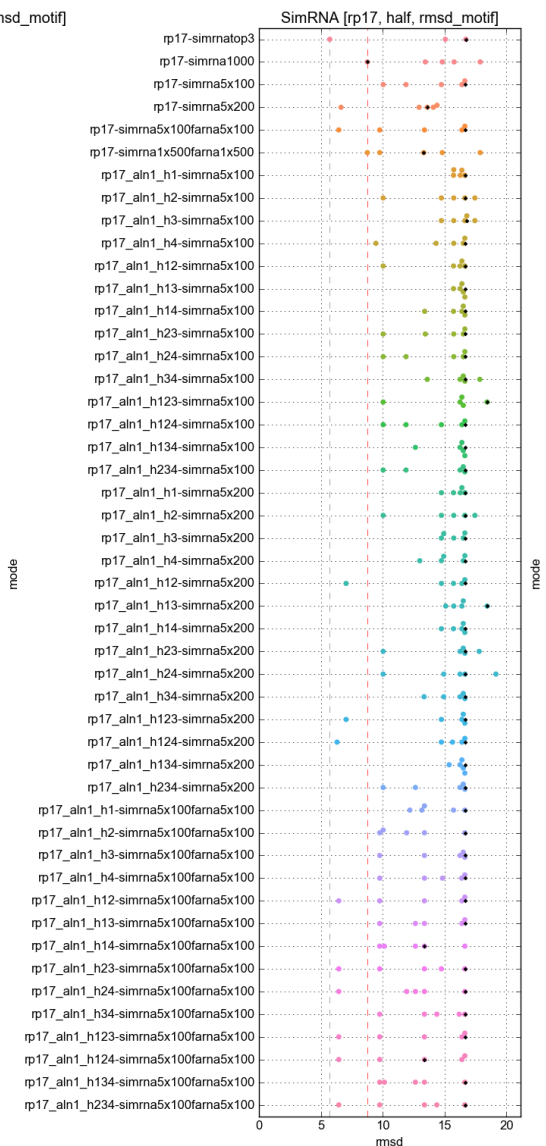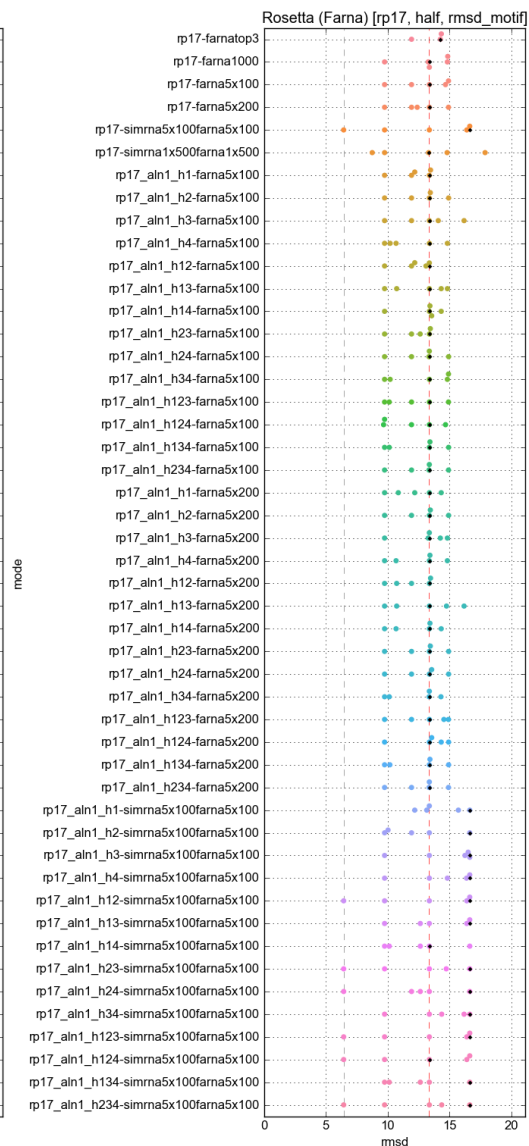

Supplement: Supplementary file 2 — Additional file 2: The analysis was performed also for various combinations of sequences of homologs (related to Fig. 6). The results of an analysis of core RMSD of all possible combinations of five input sequences of homologs for all 8 RNA families investigated in this work: Adenine riboswitch (Ade), c-di-GMP riboswitch (GMP), TPP riboswitch (TPP), THF riboswitch (THF), tRNA, RNA-Puzzle 13 (RP13), RNA-Puzzle 14 (RP14), RNA-Puzzle 17 (RP17). This analysis was performed with the evox_all_variants.py from the EvoClustRNA package. Each sequence of homologs was ordered from 1 to 3. A mode “h1” means models of the first homolog and the target sequence used for clustering, “h2” means models of the second homolog and the target sequence. “h234” means that models of three homologs were considered during clustering, the second homolog, third and fourth. For each variant 5 top clusters are shown and the first cluster is marked with a black dot. The first panel combines the results for SimRNA and Rosetta, the second panel shows the results for SimRNA and the third only for Rosetta. [file 12859_2019_3120_MOESM2_ESM.pdf]

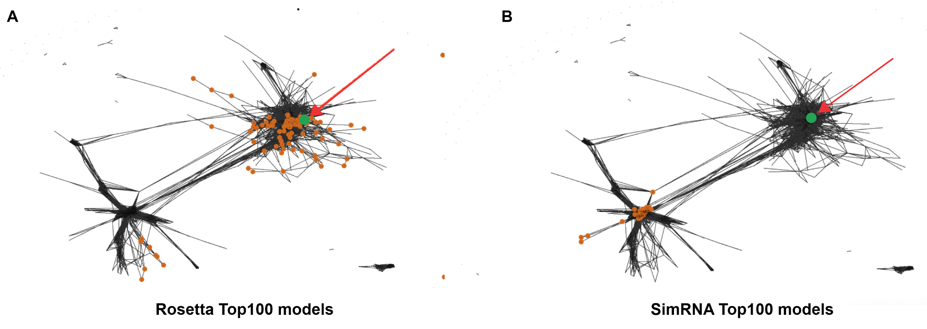

Supplement: Supplementary file 3 — Additional file 3: Figure S2. The comparison of top100 of Rosetta and SimRNA. Top 100 models from SimRNA vs Rosetta visualized with Clanstix/CLANS for models of the target sequence for the TPP riboswitch. Models obtained with (A) Rosetta and (B) SimRNA. Top 100 models from Rosetta are very different from each other and they cluster around the correct, reference structure (pointed by the red arrow). Top 100 models from SimRNA showed less diverge and cluster all altogether. [file 12859_2019_3120_MOESM3_ESM.png]

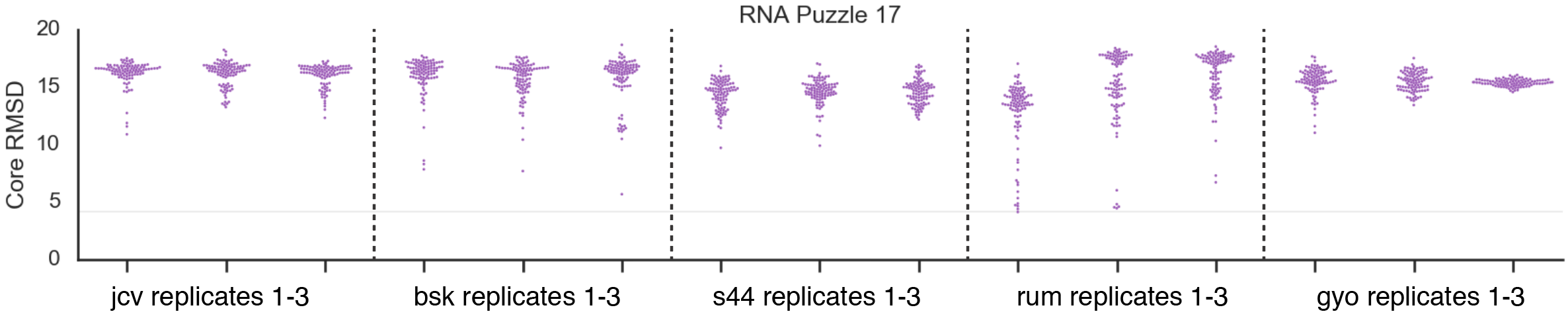

Supplement: Supplementary file 9 — Additional file 9: Figure S3. Analysis of replicates for SimRNA simulations with different initial seed values for RNA Puzzle 17. [file 12859_2019_3120_MOESM9_ESM.png]
