# Supplementary material for: RNA 3D structure prediction guided by independent folding of homologous sequences
Source: BMC Bioinformatics. 2019 Oct 22;20:512. doi: 10.1186/s12859-019-3120-y (PMC6806525; doi:10.1186/s12859-019-3120-y)
Supplement: Supplementary file 4 — Additional file 4: List of all the sequences and secondary structures used in the benchmark of EvoClustRNA and a list of links to the SimRNAweb predictions. [file 12859_2019_3120_MOESM4_ESM.pdf]

List of all the sequences and secondary structures used in the benchmark of EvoClustRNA and a list of links to the SimRNAweb predictions

ade

> 1Y26:X|PDBID|CHAIN|SEQUENCE

CGCUUCAUAUAAUCCUAAUGAUAUGGUUUGGGAGUUUCUACCAAGAGCCUUAAACUCUUGAUUAUGAAGUG

(((((((((...((((((......[[.))))))........((((((]].....))))))..)))))))))

http://genesilico.pl/SimRNAweb/jobs/ade_pk-35b2a2c1/

> AAML04000013.1

UAUAACAUAUAAUUUUGACAAUAUGGGUCAUAAGUUUCUACCGGAAUACCGUAAAUAUUCUGACUAUGUAUA

((((.((((...((.((((.....[[)))).))........(.(((((]].....))))).)..))))))))

http://genesilico.pl/SimRNAweb/jobs/9c6339e0-591c-498d-9745-1a828f9ee81d/

> BA000028.3/1103960-1104044

UUUUCAUAUAAUCGCGGGGAUAUGGCCUGCAAGUUUCUACCGGUUUACCGUAAAUGAACCGACUAUGGAAA

(((.((((...(.(((((.....[[))))).)........(((((.(]].....).)))))..)))).)))

http://genesilico.pl/SimRNAweb/jobs/7bc1d432-eac8-47cf-a42e-aa3c89efc721/

> U51115.1/15606-15691

ACCUCAUAUAAUCUUGGGAAUAUGGCCCAUAAGUUUCUACCCGGCAACCGUAAAUUGCCGGACUAUGCAGG

.(..((((...(..((((.....[[))))..)........(((((((]].....)))))))..))))..).

http://genesilico.pl/SimRNAweb/jobs/e614e4a0-0898-45f2-9964-52db07279965/

> AAFV01000199.1/524-602

((((((((...((((((....[[)))))).........(((((]]....)))))...))))))))

http://genesilico.pl/SimRNAweb/jobs/2e496700-b989-4044-883d-d34257b022ab/

tpp

> tpp

gGACUCGGGGUGCCCUUCUGCGUGAAGGCUGAGAAAUACCCGUAUCACCUGAUCUGGAUAAUGCCAGCGUAGGGAAGUUc

((((((((.((((.(((.....)))))))......)..)))).....(((...((((......))))...)))..)))))

http://genesilico.pl/SimRNAweb/jobs/16662ebf-cf31-42d1-98a3-2aae31f28087/

>CP000050.1/1019813-1019911

CCGCCGAAGUGGGGGUACCACAGCACUGCUGCGGUUGAGAUAGUCCCUUCGAACCUGAUCCGGCUCAUACCGGCGUAGGGAAGCUUCGUUAGA

UGCGCU

.....(((((((((..(((.(.........).)))........)))).....(((...((((......))))...)))...)))))......

......

http://iimcb.genesilico.pl/SimRNAweb/jobs/aed2c40b-bb70-44a7-846d-b133359fc6bd/

>BX248356.1/234808-234920

ACGAGAUGCCCGGGUGCCAUGUGCUUGCUGUACGUGGCUGAGACGGCUGUUUGGCCGAACCGUAGAACCUGAUCUGGGUAAUACCAGCGAUAG

GAAGACUUCAUACUGUGACU

.....(.(.((((..((((...............)))).....(((......))))..))).....(((...((((......))))....))

)..).).)...........

http://iimcb.genesilico.pl/SimRNAweb/jobs/0abbb76e-9cda-482f-abb2-94557e91acd8/

>AE017180.1/640928-641029

AUAGUCUGCUGGGGGAGUUCUUGGGAACUGAGACGGGCAACGCCCGAACCCUUUGAACCUGAUCCGGUUUAUACCGGCGUAGGGAAGCGGCCA

GAAACAAUC

.....(.(((.(((..((((....)))).....(((((...)))))..)))......(((...((((......))))...)))..))).)...

....

http://iimcb.genesilico.pl/SimRNAweb/jobs/6bff10d7-d4ec-43ce-8f79-8f538fa1ae65/

>AL766847.1/75304-75402

CACAAGGGAGUGCCUUGAGCUGAGAUUGCAGAUAUGCAAAAUCCUCUAACCUGAUCUCGUUAGGACGAGCGUAGGAAUUGUG

((((((((..((.....)).....(((((....)))))..))))....((((..((((......))))..))))..)))))

http://genesilico.pl/SimRNAweb/jobs/d2609d4d-bd6f-49fd-acbe-0ab278e0166b/

tRNA

>1fir

GCCCGGAUAGCUCAGUCGGAGAGCAUCAGACUUUUAAUCUGAGGGUCCAGGGUUCAAGUCCCUGUUCGGGCGCCA

(((((((..((((........))))..(((.........)))......(((((.......))))))))))))....

http://iimcb.genesilico.pl/SimRNAweb/jobs/a9bc516d-e3da-489d-93ef-5eb20e3f13c3/

>AF396436.1/4744747513

GCCGCUUGGAUGGUUCCGGUGUGGGCUCAUUUCCCAUAACUAUAAAGUUCGAUUCUUUAAAGUGGCU

(((((((...(((..)))..(((((.......))))).....(((((.......)))))))))))).

http://iimcb.genesilico.pl/SimRNAweb/jobs/822df074-320e-4166-9fd1-8fbcf085908a/

>M57527.1/170

ACUCUUAUAGCUUAAUAUUAAAGUAUAGCGCUGAAAACGCUAAGAUGAACCCUAAAAAGUUCUAGGGGUA

(((((((..((((.......)))).(((((.......)))))....(((((......)))))))))))).

http://iimcb.genesilico.pl/SimRNAweb/jobs/613bcfcf-f513-4945-9cf4-6df7db04545e/

>AB009835.1/171

CAUUAGAUGACUGAAAGCAAGUACUGGUCUCUUAAACCAUUUAAUAGUAAAUUAGCACUUACUUCUAAUGA

(((((((..(((.......)))..((((.......))))......(((.((........)))))))))))).

http://iimcb.genesilico.pl/SimRNAweb/jobs/cf61bea5-88c4-4e82-8042-dc04ce5cadcf/

>M26977.1/379453

GGGGCCAUAGGGUAGCCUGGUCUAUCCUUUGGGCUUUGGGAGCCUGAGACCCCGGUUCAAAUCCGGGUGGCCCCA

(((((((..((((...........)))).(((((.......)))))....(((((.......)))))))))))).

http://iimcb.genesilico.pl/SimRNAweb/jobs/8ca21d4d-7ceb-4736-9619-7c1814c75637/

GMP

>gmp

gCGCGGAAACAAUGAUGAAUGGGUUUAAAUUGGGCACUUGACUCAUUUUGAGUUAGUAGUGCAACCGACCGUGCUgg

((((((..((......((((((((......[[[[[[[.)))))))))...))....]]]]]..]]..))))))...

http://iimcb.genesilico.pl/SimRNAweb/jobs/faa97ed7/

>AE015927.1/474745-474827

AUUUUAAGAGGAAAUUUUGAACUAUAUACUUAUUUGGGCACUUUGUAUAUAGGGAGUUAGUAGUGCAACCGACCUUGAUUAAU

(((....((((.(((......((((((((......[[[[[[[..))))))))...)))...]]]]]..]]..))))....)))

http://genesilico.pl/SimRNAweb/jobs/e59064f8-ef9c-4c2c-864a-e20b4092cb03/

>ABFD02000011.1/154500-154585

AAAUAUUAUAGAGAUGUUGAAGUAUAUUCUAUUAUUGGGCACCUUAUGGAUAUACUGAGUCAGUGGUGCAACCGGCUAUGAAUAUA

.....((((((.(((......((((((((.......[[[[[[[....))))))))...)))...]]]]]..]]..)))))).....

http://genesilico.pl/SimRNAweb/jobs/5c0d22ec-c061-4567-aa68-3f8e5ac9ab46/

>BA000004.3/387918-388001

AAUCAAUAGGGAAGCAACGAAGCAUAGCCUUUAUAUGGACACUUGGGUUAUGUGGAGCUACUAGUGUAACCGGCCCUCCUUUAA

....(..((((.(((......((((((((.......[[[[[[[..))))))))...)))...]]]]]..]]..))))..)....

http://genesilico.pl/SimRNAweb/jobs/e5332c4d-e096-4d01-91f0-6b5ef2f92d37/

>AE000513.1/1919839-1919923

CUGUCGAAGAGACGCGAUGAAUCCCGCCCUGUAAUUCGGGCACCUCGGACGGGAGGAGCAAGUGGUGCGACCGGCUUUUCGUUGG

(((.(((((((..((......(((((.((........[[[[[[[..)).)))))...))....]]]]]..]]..))))))).)))

http://genesilico.pl/SimRNAweb/jobs/e462d8a5-7079-41df-b1bb-25edcb065cca/

THF

>thf

GGAGAGUAGAUGAUUCGCGUUAAGUGUGUGUGAAUGGGAUGUCGUCACACAACGAAGCGAGAGCGCGGUGAAUCAUUGCAUCCGCUCCA

((((....(((((((((((......(((((((...[[[[....))))))).....((....))))).)))))))))..]]]].)))).

http://genesilico.pl/SimRNAweb/jobs/7f0f8826/

>ACCL02000010.1/116901-116991

AGUAGAGUAGGUCUUAUACGUAAAGUGUCAUCGGAUGGGGAGACUUCCGGUGAACGAAGGGUUACCGCGUUAUAUGACCGCUUCCGCUACU

(((((....((((.((((......((.(((.((((..[[[[....)))).)))))...((....))....)))).))))..]]]].)))))

http://iimcb.genesilico.pl/SimRNAweb/jobs/a690ac93-1e57-4f25-9f63-aabf0700574d/

>ACKX01000080.1/10519-10620

UGCAGAGUAGAGAAUAAAGUGGUUAGUGCCCGACACACAGGGAGUUGGUGUCGAGACGAAGAGCCGAAUCGGUUCCCAGUUUUAUUUUCGCAU

CCCGCUGCC

(((((....(((((((((((.....((.((((((((...[[[[....))))))))))...((((.......))))...)))))))))))...]

]]].)))))

http://iimcb.genesilico.pl/SimRNAweb/jobs/cb6e7e4d/

> haq

UGCAAAAUAGGUUUCCAUGCGUCAAGUGUUUUGUGGAUGGGGAGUUGCCACAGAAACGAAAAGUCGGUUCGCGUGCGGACCGGACUUACGAUA

UGGUUACCGCACCCGUUGCA

(((((....(((..(((((......((.(((((((((...[[[.....))))))))))))...(((.....................)))....))

)))..))))...]]].)))))

http://genesilico.pl/SimRNAweb/jobs/497811c4/

> hcp

GGUAGAGUAGGUGUCUCGCGUUAAGUGCCAAGGGAUGGGACGUUGCCCUUGGACGAAAGCUAUUAAGAGCUGCGUUGGGACAUCGCGUUCGCU

AUC

(((((....((((((((((.....((.(((((((...[[[[....)))))))))...((((......))))...))))))))))..]]]].))

)))

http://iimcb.genesilico.pl/SimRNAweb/jobs/fae110a9/

RNA-Puzzle 13

> zmp

gggucgugacuggcgaacaggugggaaaccaccggggagcgaccccggcaucgauagccgcccgccugggc

(((((((....[[[[....(((((....))))).....)))))))...........(((...]]]]..)))

http://genesilico.pl/SimRNAweb/jobs/175dd34c-100b-4a46-9aaa-e773b1468c39/

>CU234118.1/352539-352459

gcucucgcgcgacuggcgacuuuggauggagcaccaucggggagcgcgggaucgaccgccgugcgccugggc

((((((((((....[[[[......(((((....))))).....))))))))))....(((...]]]]..)))

http://genesilico.pl/SimRNAweb/jobs/0bf5c25e-4936-4da7-b145-928eea4031c7/

>BAAV01000055.1/28972982

ugaguuuucugcgacugacggauuauugcagagcacugcaagggaacagaaaaacucuuuuucagccgaccgucugggcacaccug

....(((((((.....[[[[[.....(((((....))))).......)))))))...........(((..]]]]]..))).......

http://genesilico.pl/SimRNAweb/jobs/8a418378-29f5-45df-af4a-5ecac1a5e7a4/

>CP000927.1/5164264-5164343

gcccguucgcgugacuggcgcuagugauggggaaccaucggggagcgcgaaccacaucgccgcgcgccugggcuccucga

....((((((((....[[[[[....(((((....))))).....))))))))......(((..]]]]]..))).......

http://genesilico.pl/SimRNAweb/jobs/d1969c5d-5a55-4025-944e-089de20719cf/

> AP009385.1/718103-718202

ucaccccugcgugacuggcgauagaacccucggguucaagguggagcaucccaccgugaagcgcagggcgccguuuuugccguucgccugggc

agccguu

....((((((((....[[[[[..(((((....)))))..(((((......))))).....))))))))........(((((..]]]]]..)))

)).....

http://genesilico.pl/SimRNAweb/jobs/9ca56ed4-69bb-477b-8ac2-35bfd085685f/

RNA-Puzzle 14

>rp14

CGUUGACCCAGGAAACUGGGCGGAAGUAAGGCCCAUUGCACUCCGGGCCUGAAGCAACGCG

(((((.(((((....))))).........((((((..........))))))....)))))..

http://genesilico.pl/SimRNAweb/jobs/1aa9a03c-33e4-4718-899e-54ab3158d64c/

>aj6 AJ630128.1

AUCGUUCAUUCGCUAUUCGCAAAUAGCGAACGCAAAAGCCGACUGAAGGAACGGGAC

..(((((.((((((((........)))))))).......((....))....))))).....

http://genesilico.pl/SimRNAweb/jobs/r14aj63pk-2f5f0e3d/

>cy2 AACY023015051.1

CGUUCAUCUUAUUUUAUUAAAUAGGACGGAAGUAGGAAGAUAGGAAAACCUCUUUCUUUUUUAAAGAAAGGCUAGCAAGUACCGCUUGGGUUA

AUUUAUCUUAGGCGGGAACGAGACCGAAUAUCUGCCGAAGGAACGC

(((((.(((((((......)))))))..[.....((..((((((....)).((((((((...))))))))(((....))).(((((((((...

......)))))))))...((....))..))))..))....)))))).

https://iimcb.genesilico.pl/SimRNAweb/jobs/r14aacy23+m+pk2-84f4be23/

>AACY020096225.1 aa20

UACGUUCAUCAUCCGUUUGGAUGACGGAAGUAAGCGAAAGCUGAAGGAACGCAUG

..(((((.((((((....))))))..[.....(((....)))....)))))]...

https://iimcb.genesilico.pl/SimRNAweb/jobs/rp14_aa20-a52d7ba5/

>a22 AACY022736085.1/436-491

UUCGUUCAUCUUACCCUGUAAGACGGAAGUAGACAUUCUUGUCGAAGGAACGCAUA

..(((((.((((((...))))))..[.....((((....))))....)))))]...

https://iimcb.genesilico.pl/SimRNAweb/jobs/rp14_aa22-6d8fb934/

RNA-Puzzle 17

>rp17

CGUGGUUAGGGCCACGUUAAAUAGUUGCUUAAGCCCUAAGCGUUGAUAAAUAUCAGGUGCAA

((((..[[[[[.))))........((((.....]]]]]....(((((....)))))..))))

http://iimcb.genesilico.pl/SimRNAweb/jobs/27b5093d/

>hcf

UGCCGUUUGAGCGGCAUUAAACAGGUCUUAAGCUCAAAGCGUCACCGCCUACAAUGCUAGGCGGUGGGUGACA

((((..[[[[[.))))........(((.....]]]]]....((((((((((......))))))))))..))).

http://genesilico.pl/SimRNAweb/jobs/6d8062dd/

>s223

GCUCGUCUGGGCGAGGAUAAAUAGCUGUUAGGCCCAGAGCGGCUCUUCGGAUUGUGUUCCCUCCGCAAUCCGGGGAGCGUCAGC

.(((..[[[[[.)))........((((.....]]]]]....(((((((((((((((.......)))))))))))))))..))))

http://genesilico.pl/SimRNAweb/jobs/36828e10/

>s221

AGCCGUUGCGGCGGCUAUAAAUAGGACAUUAAGCCGCAAGCGUUGCCCGGUAUACCGCCGGGCAGGUUGUC

((((..[[[[[.))))........((((.....]]]]]....(((((((((.....)))))))))..))))

http://genesilico.pl/SimRNAweb/jobs/742b47e6/

>pisol

AGCCGUUCGGGCGGCUAUAAACAGACCUCAGGCCCGAAGCGUGGCGGCGCCGCCGGUGGUA

((((..[[[[[.)))).......((((.....]]]]]....((((((()))))))..))))

http://genesilico.pl/SimRNAweb/jobs/336e0098/
